# Yuye Jinhua Qingre Tablets Attenuate Acute Pharyngitis by inhibiting the Complement Cascade and C5a/C5aR1 Axis

**DOI:** 10.1186/s13020-025-01191-1

**Published:** 2025-08-25

**Authors:** Yifei Gao, Leiming You, Jiying Zhou, Xiaoyu Tao, Zhengsen Jin, Chao Wu, Fanqin Zhang, Siyu Guo, Haojia Wang, Yueqin Guan, Hua Luo, Jiarui Wu

**Affiliations:** 1 School of Chinese Materia Medica, Beijing University of Chinese Medicine, Beijing, 102488 China; 2 School of Life Science, Beijing University of Chinese Medicine, Beijing, 102488 China; 3 Jiuhua Huayuan Pharmaceutical Co., Ltd., Chuzhou, 239004 China; 4 College of Pharmacy, Guangxi Medical University, Nanning, 530021 China

**Keywords:** Yuye Jinhua Qingre Tablets, Acute pharyngitis, Complement pathway, C5a/C5aR1 axis

## Abstract

**Background:**

Acute pharyngitis (AP) is a common upper respiratory tract infection, primarily characterized by symptoms such as throat pain, redness, swelling, and difficulty swallowing. It is typically caused by viral infections, bacterial infections, or physical and chemical irritants. Yuye Jinhua Qingre Tablets (YYJH) are recognized for their ability to clear heat, detoxify, reduce swelling, and alleviate pain, making them a common treatment option for acute pharyngitis. However, research on their specific mechanisms of action is still inadequate.

**Methods:**

Using UPLC-Q-Exactive-Orbitrap-MS technology combined with serum pharmacochemical analysis, the main chemical components and blood components of YYJH were identified. The anti-inflammatory activity was verified through the ammonia-induced acute pancreatitis (AP) model in SD rats and the LPS-stimulated NP69SV40T cell inflammation model. Integrating transcriptomics, proteomics, and bioinformatics analysis revealed the mechanism of YYJH in treating AP, which was further validated by molecular biology experiments.

**Results:**

Twelve blood-entry components were identified, and their anti-inflammatory effects were validated using the SD rat acute pancreatitis (AP) model and the NP69SV40T cell inflammation model. The study results indicated that the drug significantly improved the pathological damage of the pharyngeal mucosa in rats with the AP model, reducing the levels of inflammatory cells in peripheral blood and serum inflammatory factors. The combined analysis of transcriptomics and Astral DIA proteomics revealed that the anti-inflammatory effects of YYJH are associated with the regulation of the classical complement pathway, characterized by the downregulation of complement components C1q, C3, C5, C9, and the modulation of macrophage infiltration and pro-inflammatory cytokine release through the C5a/C5aR1 axis. Gene set enrichment analysis further suggested that YYJH can alleviate AP-related metabolic disorders and immune dysregulation. Molecular biology experiments demonstrated that after YYJH intervention, the complement cascade reaction was significantly inhibited, with downregulated expression levels of C5a and C5aR1, and decreased membrane localization signals of the macrophage marker F4/80, along with reduced expression levels of inflammatory factors.

**Conclusions:**

Research indicates that YYJH exerts anti-inflammatory effects by regulating the classical complement pathway and the C5a/C5aR1 axis, inhibiting the production of inflammatory mediators and the activation of immune cells. This provides a theoretical basis for the molecular mechanisms underlying traditional Chinese medicine in the treatment of acute pharyngitis.

**Graphical Abstract:**

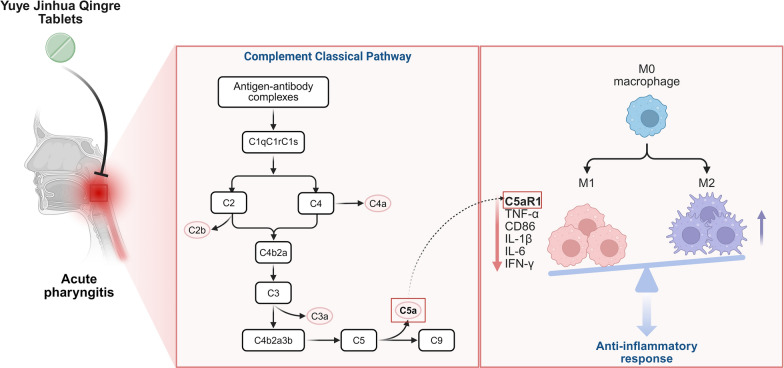

**Supplementary Information:**

The online version contains supplementary material available at 10.1186/s13020-025-01191-1.

## Introduction

Acute pharyngitis (AP) is a common inflammatory disease among upper respiratory tract infections, characterized by congestion and edema of the pharyngeal mucosa, infiltration of inflammatory cells, and excessive release of pro-inflammatory factors [1]. Despite the widespread clinical use of antibiotics and glucocorticoids, issues such as antibiotic resistance, side effects, and high recurrence rates remain to be addressed [2–4]. Traditional Chinese medicine (TCM) formulations exhibit unique advantages in regulating immune homeostasis and inhibiting inflammatory cascades due to their multi-component synergistic effects [5, 6]. The main components of Yuye Jinhua Qingre Tablets (YYJH) include *Mussaenda Pubescens* (Yuye Jinhua), *Andrographis Paniculata* (Chuanxinlian), *Gardenia Jasminoides* (Zhizi), *Achyranthes Aspera* (Daokoucao), *Scrophularia Ningpoensis* (Xuanshen), and artificial bezoar (Rengong Niuhuang), which possess heat-clearing, detoxifying, and swelling-reducing analgesic effects. As a TCM preparation for the clinical treatment of acute pharyngitis, studies have confirmed its high overall effective rate for acute pharyngitis with wind-heat syndrome, effectively alleviating symptoms such as sore throat, dry burning sensation in the throat, redness and swelling of the pharyngeal mucosa and uvula, and lymphoid follicle hyperplasia on the posterior pharyngeal wall [7–9]. However, the pharmacological basis and molecular mechanisms of its efficacy have yet to be systematically elucidated. In recent years, with the development of systems biology technologies, transcriptomics and proteomics have provided important tools for elucidating the "multi-target-multi-pathway" action mode of TCM formulations [10–12].

The complement system plays a crucial role in the immune defense and regulation processes of the human body. The classical pathway of complement, as a core effector mechanism of the innate immune system, is closely associated with the pathological progression of inflammatory diseases such as rheumatoid arthritis due to its abnormal activation [13]. The recognition of antigen–antibody complexes mediated by C1q triggers a cascade of C4/C2 cleavage reactions, leading to the generation of C3 convertase (C4b2a) and the formation of the membrane attack complex (MAC). This process plays a key role in typical inflammatory responses, such as mucosal congestion in the pharynx and lymph follicle hyperplasia [14]. The signaling axis formed by complement anaphylatoxin C5a and its receptor C5aR1 (C5a/C5aR1 axis) mediates immune cell chemotaxis, pro-inflammatory factor release, and tissue damage, making C5a an important target for anti-inflammatory therapies [15].

However, traditional drugs typically intervene in the complement system at a single point, while traditional Chinese medicine formulations may achieve systemic regulation through the synergistic action of multiple components. This hypothesis still requires experimental validation. Although existing studies have revealed the anti-inflammatory phenotype of Yuye Jinhua Qingre Tablets, the regulatory mechanisms of this formulation on the classical pathway of the complement system have not yet been clarified.

This study aims to systematically reveal the mechanism of action of Yuye Jinhua Qingre Tablets in the intervention of acute pharyngitis through the integration of material basis analysis, in vitro and in vivo pharmacodynamic evaluation, and multi-omics technology. The research first characterizes the blood components using UPLC-Q-Exactive-Orbitrap-MS technology, identifying 12 blood-entry components, including clerodane-type diterpenoids and bile acids. An acute pharyngitis model was constructed in rats induced by ammonia, and the anti-inflammatory efficacy of the drug was verified through pathological, hematological, and cytokine assays. Furthermore, transcriptomic sequencing and proteomics were utilized to analyze the functional enrichment characteristics of differentially expressed genes and proteins, constructing a "gene-protein-pathway" interaction network. Special attention was paid to the dynamic regulation of the classical complement pathway and the C5a/C5aR1 axis, elucidating the multi-level effects of the drug on immune cell polarization, inflammatory mediator release, and mucosal repair. The results reveal that Yuye Jinhua Qingre Tablets achieves a "anti-inflammatory-repair" synergistic effect by inhibiting the complement cascade, blocking C5a/C5aR1 signal transduction, and regulating the pro-inflammatory phenotype transformation of macrophages, providing a theoretical basis for the molecular mechanisms of Traditional Chinese Medicine in treating acute pharyngitis.

## Materials and methods

### Identification of the main chemical components and blood-entry components of Yuye Jinhua Qingre Tablets (YYJH)

YYJH (batch numbers: 220701) was supplied by Jiuhua Huayuan Co., Ltd. (Jiangxi, China). Ultra Performance Liquid Chromatography-Quadrupole-Exactive Orbitrap Mass Spectrometry (UPLC-Q-Exactive-Orbitrap-MS) was performed on a Waters ACQUITY UPLC BEH-C18 analytical column (150 mm × 2.1 mm, 1.7 μm, Milford, MA, United States). The oven was set at 30 °C; the injection volume was 5 μL; the flow rate was set at 0.2 mL·min^−1^; the mobile phase was consisted of methanol (A) and 0.1% formic acid in water (B). The elution program was as follows: 10% A for 0–27 min, 57%-95% A for 27–50 min, 95% A for 50–54 min, 95%-10% A for 54–55 min, and 10% A for 55–60 min. The ion source used was electrospray source ionization (ESI). Mass spectrometry (Thermo Fisher Q-Exactive Plus) was performed in both positive and negative ion modes. Spray voltage: 3.5 kV; Capillary temperature: 320 ℃; Auxiliary gas temperature: 400 ℃; Collision energy: 20, 40, 60 eV; Detection method: Full MS/dd-MS^2^ (Full MS resolution of 70,000, dd-MS^2^ resolution of 17,500); Quality scanning range: m/z: 100 ~ 1500; S-Lens RF Level: 55.

### In vivo* experiments*

**Fig. 1:**
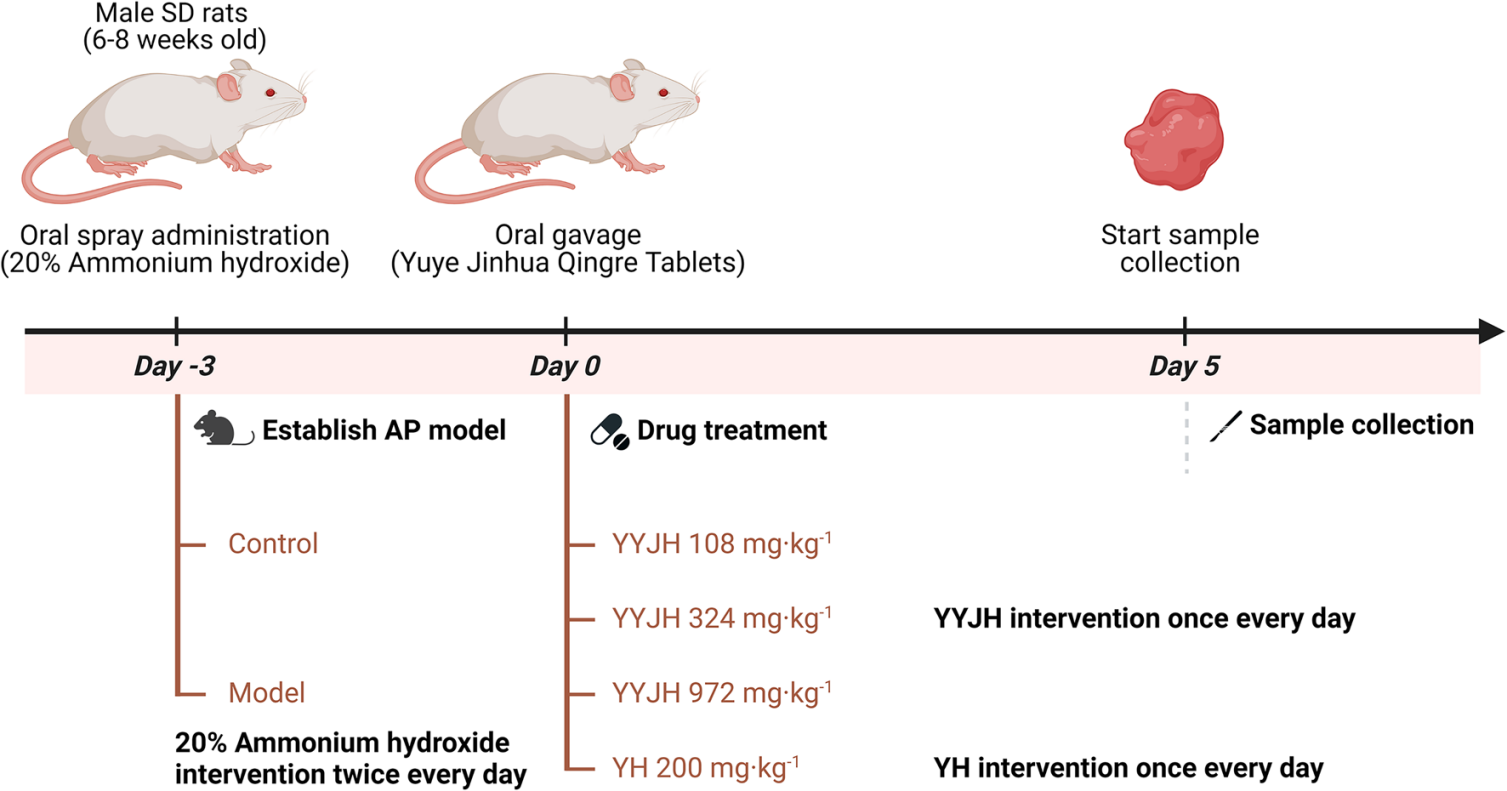
Schematic diagram of experimental protocol for YYJH treatment of AP rats

In the experiment involving the formation of AP, male SD rats (6–8 weeks old, weighing 280–300 g, animal certificate number: SCXK 2023–0011) were purchased from the Beijing Huafukang Biotechnology Co., Ltd (Beijing, China), and they were kept in an SPF environment with experimental animal welfare care. As shown in Fig. 1, 90 SPF grade SD rats were adaptively fed for one week and randomly divided into 6 groups, with 15 rats in each group, the normal group was given the same amount of distilled water, and the other groups prepared the AP model of rats according to the throat mucous membrane ammonia spray stimulation in the specification for the preparation of AP animal models (draft). The specific operation was: 20% ammonia was sprayed into the throat spray of rats in each group with ear nose throat anesthesia spray tube, 0.02 ml each time, once in the morning and once in the afternoon, for three consecutive days, the throat mucous membrane of rats was congested and swollen due to acute stimulation, forming acute inflammation, and the AP rat model was established. The successfully modeled rats were randomly divided into 5 groups, including the model group (Model), the positive drug Yinhuang granules group (YH 200 mg kg^−1^), the low-dose group of Yuye Jinhua Qingre Tablets (YYJH 108 mg kg^−1^), the medium dose group of Yuye Jinhua Qingre Tablets (YYJH 324 mg kg^−1^), and the high-dose group of Yuye Jinhua Qingre Tablets (YYJH 972 mg kg^−1^). The corresponding doses of drugs were administered by gavage daily, and the normal group (Control) was given an equal amount of distilled water for 5 consecutive days. Relevant experimental indicators were tested.

At 0.5, 1, and 2 h after the last administration, orbital blood samples were collected from all rats in the model group, low, medium, and high dose groups of Yuye Jinhua Qingre Tablet, totaling 4 groups, at 3 time points for identification of blood components. After the completion of orbital blood collection, the rats in each group were fasted without water for 12 h, anesthetized with pentobarbital sodium (intraperitoneal injection), and blood was collected from the abdominal aorta. 500 μ L of whole blood was placed in an EDTA anticoagulant tube for complete blood count (CBC), and the remaining whole blood was placed in a vacuum blood collection tube. Subsequently, serum was collected for ELISA detection. Immediately peel off the pharyngeal mucosa and submucosal tissue of rats, wash the tissue with physiological saline, and dry it with filter paper. Partially fixed with 4% PFA for 48 h, subjected to H&E staining, immunohistochemistry (IHC) staining, and immunofluorescence (IF) staining; Partially frozen in liquid nitrogen and stored at -80 ℃ for RT qPCR and Western blot analysis. This animal experiment has been approved by the Animal Ethics Committee of Beijing University of Traditional Chinese Medicine (Ethics number: BUCM-2023113001–4147).

### Hematoxylin–eosin staining (H&E), Immunohistochemistry (IHC), Immunofluorescence staining (IF), Blood routine test, and enzyme-linked immunosorbent assay (ELISA)

The rat pharyngeal tissues were fixed in 4% paraformaldehyde (PFA) for 48 h, embedded in paraffin and sectioned. Incubation with specific primary antibodies (Servicebio, China; Bioss, China), incubation with secondary antibodies, washing, staining with DAB reagent (Abcam, USA), and counterstaining with hematoxylin, to obtain the final IHC sections. Double staining the cell nucleus with DAPI, quenching with spontaneous fluorescence, and sealing with anti fluorescence quenching agent to obtain IF slices.

The number of various inflammatory cells in the blood, including white blood cells, neutrophils, lymphocytes, and monocytes, was detected using a five class hemocytometer.

According to the manufacturer's instructions, the Rat TNF-α ELISA kit (Elabscience, China, E-EL-R2856c), Rat IL-6 ELISA kit (Elabscience, China, E-EL-R0015c) was used for ELISA detection of serum samples, and the Human TNF-α ELISA kit (Elabscience, China, E-EL-H0109), Human IL-6 ELISA kit (Elabscience, China, E-EL-H6156) was used for ELISA detection of cell supernatant, and the Rat complement component 3a (C3a) ELISA kit (Elabscience, China, E-EL-R0255, Rat complement component 5a (C5a) ELISA kit (Elabscience, China, E-EL-R3011) was used for ELISA detection of rat pharyngeal tissue samples.

### Cell lines, culture conditions and drug treatment

Human nasopharyngeal epithelial cell lines NP69SV40T was purchased from Procell Life Science & Technology Co., Ltd. (Wuhan, China). The cell lines were authenticated by STR profiling and verified to be free of mycoplasma contamination. NP69SV40T cells were cultured in specialized culture medium (Procell, China). The cells were cultured in a saturated humidity environment at 37 °C and 5% CO_2_.

### Cell counting kit-8 (CCK-8) assay

The Cell Proliferation and Cytotoxicity Test Kit (Dojindo, Japan) was utilized in accordance with the manufacturer’s protocol for the YYJH efficacy experiment. Briefly, 2 × 10^4^ cells was seeded per well in 96-well plates. After YYJH intervention, a CCK-8 Assay Kit (Dojindo, Japan) was used to detect the proliferation ability of the Human nasopharyngeal epithelial cells. In brief, 10 μL of CCK-8 reagent was added directly to the cell culture medium per well, and the Human nasopharyngeal epithelial cells were routinely incubated for 2–4 h in a cell culture incubator. Subsequently, the absorbance was measured using a SpectraMax i3x microplate reader (Molecular Devices, USA) at a wavelength of 450 nm. These experiments were carried out independently three times.

### Reverse transcription quantitative polymerase chain reaction (RT-qPCR) analysis

An RNA Easy Fast Cell Kit (Tiangen, China) was used for total RNA isolation according to the manufacturer’s instructions. The quality of the total RNA was determined by a SpectraMax Quick Drop reader (Molecular Devices, USA). Total RNA (1 μg) was used for cDNA synthesis of mRNA following the instructions of the ReverTra Ace qPCR RT Kit (Toyobo, Japan). Quantitative polymerase chain reaction (qPCR) was performed to measure the relative expression of target genes, using SYBR Green Realtime PCR Master Mix (Toyobo, Japan) with the QuantStudio 6 Flex (Applied Biosystems, USA). GAPDH was used as a control and the 2^−ΔΔCt^ method was used for the data analysis. The primers for the target genes were synthesized by Sangon Biotech Co., Ltd. (Shanghai, China) and their sequences are listed in the Supplementary Methods.

### Western blot

Rat pharyngeal tissues was collected in RIPA lysis buffer supplemented with Protease and Phosphatase Inhibitor Cocktail (Beyotime, China). The collected samples were then centrifuged at 13,000 rpm and 4℃ for 10 min. The supernatants were preserved and used for western blot analysis. The total protein concentration was measured with a BCA Protein Assay Kit (Solarbio, China). Total protein (20 µg) was mixed with 5 × sample buffer, boiled at 99 ℃ for 5 min and loaded onto 10% SDS-PAGE gels. Then the protein bands were transferred onto NC membranes and blocked with 5% nonfat milk for 2 h at room temperature. The NC membranes with proteins were incubated with diluted primary antibodies (ABclonal, Affinity or Proteintech, China) at 4℃ overnight, including anti-C9 (Servicebio, China, GB112622-100, 1:500), anti-C1QB (ABclonal, China, A5339, 1:1000), anti-C1R (ABclonal, China, A15650, 1:1000), anti-C1S (ABclonal, China, A6878, 1:1000), anti-C2 (ABclonal, China, A10186, 1:1000), anti-C3 (Affinity, China, DF13224, 1:500), anti-C4 (Proteintech, China, 22233-1-AP, 1:3000), anti-C5 (Affinity, China, DF7719, 1:1000), anti-CD86 (ABclonal, China, A19026, 1:1000), anti-CD68 (ABclonal, China, A13286, 1:1000), anti-IFN-γ (Proteintech, China, 10808-1-AP, 1:500), anti-IL-1β (Proteintech, China, 26048-1-AP, 1:1000), anti-TNF-α (Proteintech, China, 60291-1-Ig, 1:1000), anti-IL-6 (Proteintech, China, 66146-1-Ig, 1:1000), and anti-GAPDH (1:5000) antibodies. Then, the membranes were incubated with relative sources of secondary antibodies (1:5000) at room temperature for 2 h. Finally, the specific protein bands were visualized with Immobilon Western Chemiluminescent HRP Substrate (Millipore, Sigma, USA). ImageJ software was used for image analysis and the signals of specific proteins were normalized to those of GAPDH.

### Transcriptomics analyses

Perform transcriptomic analysis using eukaryotic transcriptome analysis. Take pharyngeal tissue samples from normal group (NC), model group (MX), and the low-dose group of Yuye Jinhua Qingre Tablets (L), the medium dose group of Yuye Jinhua Qingre Tablets (M), and the high-dose group of Yuye Jinhua Qingre Tablets (H) rats. After RNA extraction, identification, and quantification of three replicate samples in each group, perform on machine sequencing using Illumina Novaseq 6000/MGISEQ-T7 sequencing platform. Use t-test to calculate FC and p-values to identify DEGs. The threshold for DEGs is set to | FC |≥ 1.5, with a *p*-value < 0.05.

### Proteomics analyses

Pharyngeal tissue samples from rats in the normal group (NC), model group (MX), and the low-dose group of Yuye Jinhua Qingre Tablets (L), the medium dose group of Yuye Jinhua Qingre Tablets (M), and the high-dose group of Yuye Jinhua Qingre Tablets (H) were subjected to tandem mass tag (TMT) labelling proteomics. Proteome Discoverer 1.4 software (Thermo Fisher Scientific, USA) was performed for identification and quantitation analysis. DEPs were identified using a *t*-test to calculate the FC and *p* value. The threshold for DEPs was set at a |FC| of ≥ 1.5 and a *p* value of < 0.05.

### Bioinformatics analysis

The Gene Ontology (GO, http://geneontology.org/) database, the Kyoto Encyclopedia of Genes and Genomes (KEGG, https://www.kegg.jp/), and the Hallmark gene set of the MSigDB (https://www.gsea-msigdb.org/gsea/msigdb) database were used to perform enrichment analysis of the biological process (BP), cellular component (CC), molecular function (MF), pathway and other biological entries for all differentially expressed genes (DEGs) and differentially expressed proteins (DEPs). The above procedures were implemented using the clusterProfiler package within R software based on the hypergeometric distribution algorithm. *P* value < 0.05 were considered to indicate statistical significance. The Search Tool for the Retrieval of Interacting Genes/Proteins (STRING, https://string-db.org/) was utilized to procure protein–protein interactions (PPI) data. The PPI network were constructed using Cytoscape software (version 3.9.1). The differential analysis between the two groups was conducted using the limma package in R software.

### Statistical analysis

All of the data were statistically analyzed using R software (version 4.2.1) or GraphPad Prism 9.0 software, and the data are presented as *mean* ± *SD*, unless otherwise stated. Independent Samples *t*-test was used to compare the differences between the two groups. One-way analysis of variance (one-way ANOVA) and Tukey's honest significant difference (Tukey's HSD) test were used to compare the differences between groups. A *p* value less than 0.05 was considered to indicate statistical significance.

## Results

### A total of twelve blood-entry components were identified in YYJH

**Fig. 2:**
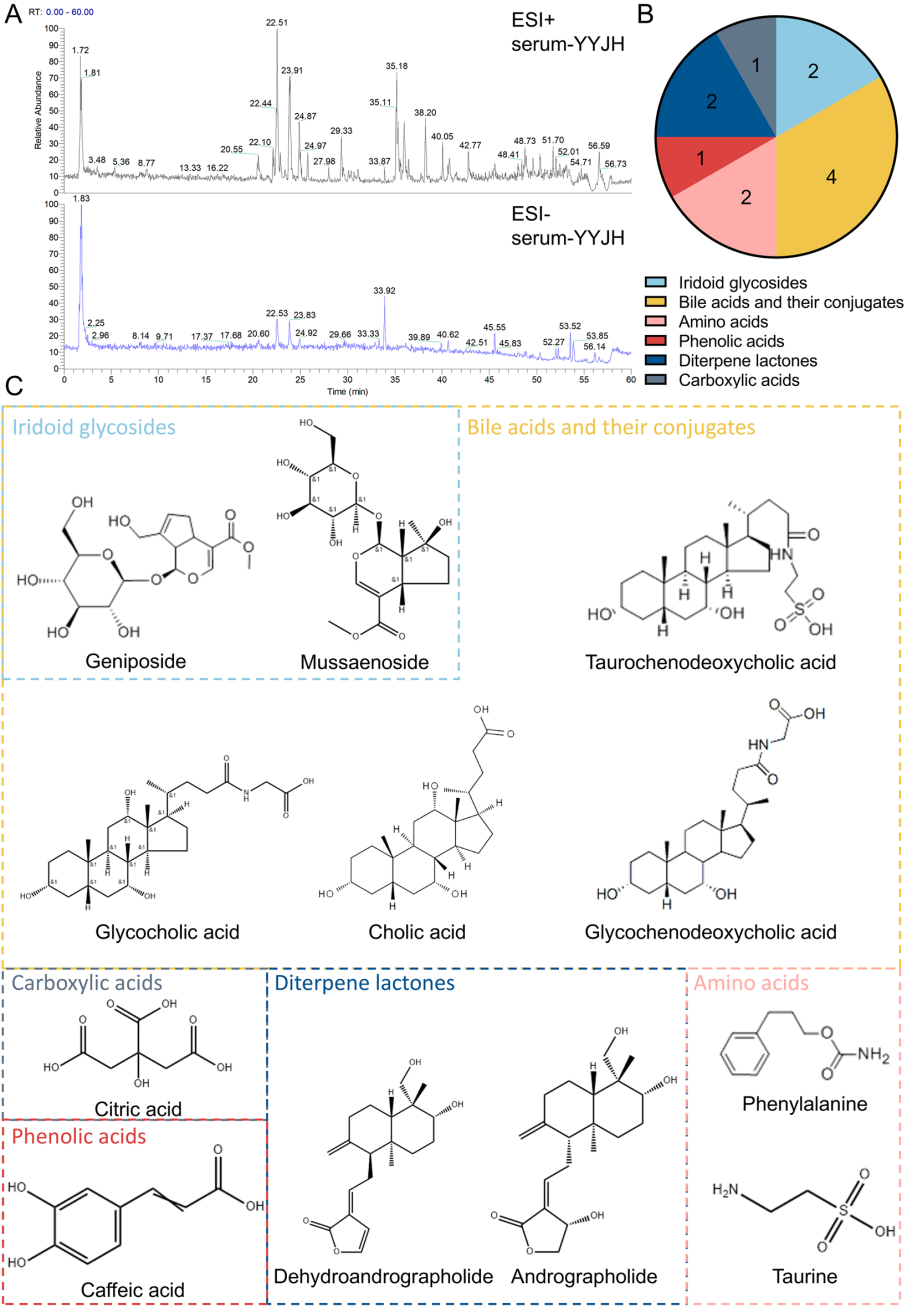
Twelve blood-entry components were identified in YYJH. **A** Total ion chromatogram (TIC) of drug containing serum; **B** Classification and statistical chart of 12 constituents absorbed into blood; **C** The chemical structural formulas of 12 constituents absorbed into blood

Using Thermo Xcalibur Qual Browser software, the mass spectrometry data of drug containing serum, blank serum, and the test solution of Yuye Jinhua Qingre Tablets were analyzed. Based on the retention time, mass to charge ratio, and mass spectrometry fragmentation pattern of each component, combined with literature information, a total of 60 drug components were detected. Classified by compound structure, it includes 16 iridoid glycosides, 5 flavonoids, 7 bile acids, 3 terpenes and steroids, 11 organic acids and their derivatives, 10 andrographolides and related derivatives, 2 amino acids and small molecule metabolites. Twelve blood-entry components were characterized and identified, including geniposide, methyl jasmonate, glycocholic acid, phenylalanine, caffeic acid, glycodeoxycholic acid, taurodeoxycholic acid, andrographolide, dehydrated andrographolide, taurine, citric acid, and cholic acid (Table 1; Fig. 2).

**Table 1:** Blood-entry components in YYJH

Mode	No	Compound name	Formula	*t*_R_ (min)		Calculated (*m/z*)		Observed (*m/z*)	Mass error (ppm)	MS/MS
[M + H]^+^	**1**	Geniposide	C_17_H_24_O_10_	14.45		387.1285		387.1282	-0.7	355.1044, 341.1093, 225.0768, 207.0661, 147.0443, 127.0389, 123.0441, 119.0339, 101.0232
[M + H]^+^	**2**	Mussaenoside	C_17_H_26_O_10_	41.56		391.1598		391.1590	-2.1	373.27289, 355.26410, 159.11714, 119.03310, 98.22581, 74.99190, 68.84775
[M + H]^+^	**3**	Glycocholic acid	C_26_H_43_NO_6_	42.06		466.3163		466.3151	-2.6	430.2940, 412.2855, 353.3494, 337.2532, 272.0925, 227.1812, 213.1644, 178.6233, 145.1006, 89.4083, 76.0403, 68.6482
[M + H]^+^	**4**	Phenylalanine	C_10_H_13_NO_2_	4.89		166.0862		166.0865	1.8	209.7565, 149.0605, 131.0495, 120.0812, 104.9638, 90.9485, 70.0021, 62.1227
[M + Na]^+^	**5**	Glycochenodeoxycholic acid	C_26_H_43_NO_5_	24.59		472.3033		472.3014	-4.0	455.2853, 404.7690, 224.1874, 133.0864, 89.0604, 68.6326
[M + Na]^+^	**6**	Taurochenodeoxycholic acid	C_26_H_45_NO_6_S		53.43	522.2859	522.2862		0.6	504.3478, 339.2904, 258.1114, 238.4904, 184.0736, 166.0636, 125.0001, 104.1075, 98.9848, 86.0971
[M + H_2_0 + H]^+^	**7**	Andrographolide	C_20_H_30_O_5_		34.1	333.2060	333.2062		0.6	315.1953, 297.1851, 285.1863, 257.1536, 203.1066, 172.1304, 133.1014, 121.1012, 107.0863, 93.0706, 79.8600, 69.3162
[M + H_2_0 + H]^+^	**8**	Dehydrated andrographolide	C_20_H_28_O_4_		34.05	315.19554	315.1957		0.5	297.1868, 285.1861, 257.1552, 241.1230, 203.1077, 163.0759, 159.1174, 121.1017
[M − H]^−^	**9**	Taurine	C_2_H_7_NO_3_S		1.91	124.0062	124.0062		0.0	136.13356, 124.99945, 79.95600, 78.95787, 62.96258
[M − H]^−^	**10**	Citric acid	C_6_H_8_O_7_		1.98	191.0186	191.0193		3.6	173.0084, 160.8413, 146.0235, 133.0133, 115.0023, 111.0076, 101.0230, 87.0075, 85.0285, 69.9971, 57.0332
[M − H]^−^	**11**	Cholic acid	C_24_H_40_O_5_		45.48	407.2792	407.2811		4.7	389.26950, 363.29068, 343.26630, 325.25500, 289.21866, 254.15192, 193.15935, 148.92697, 129.97130, 95.04901, 68.80843
[M − H]^−^	**12**	Caffeic acid	C_9_H_8_O_4_		25.2	179.0338	179.0347		5.0	150.9530, 134.9869, 128.1606, 121.0284, 98.2357, 90.9968, 75.2433, 70.3967, 66.1907

### YYJH can ameliorate the pathological phenotype of AP and exert anti-inflammatory effects

**Fig. 3:**
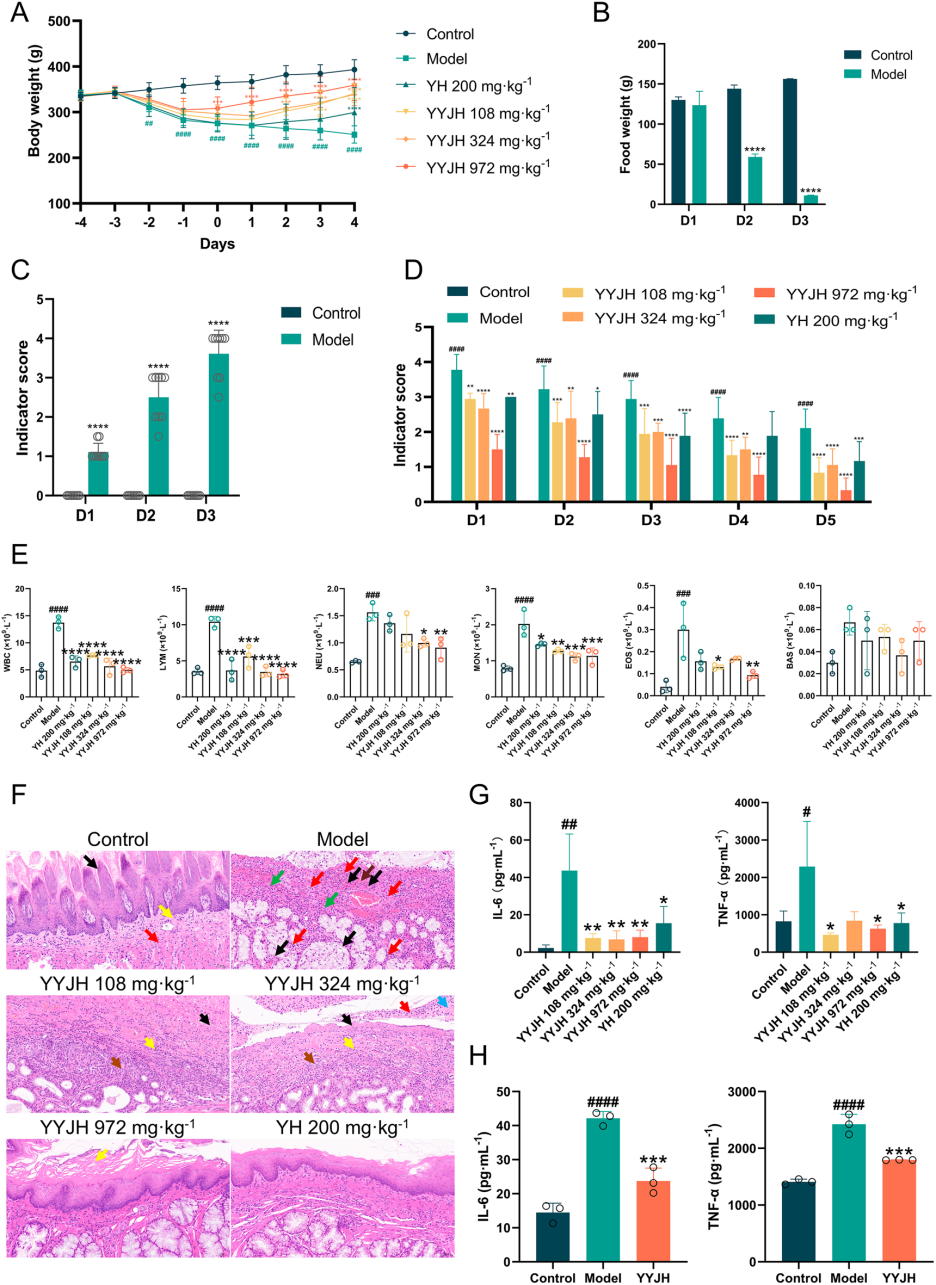
YYJH intervenes in multiple pathophysiological indicators in vivo. **A** Changes in body weight of rats across each group throughout the experiment, *n* = 9; **B** Food intake of rats in each group during the 3-day modeling period, *n* = 3; **C** Apparent index scores of rats in each group during the 3-day modeling period, *n* = 9; **D** Apparent index scores of rats in each group following 5 days of drug administration, *n* = 9; **E** Number of white blood cells and differential counts in the blood of rats in each group, *n* = 3; **F** H&E staining results of pharyngeal tissue in rats from each group (bar = 50 μm), *n* = 6; **G** Changes in serum inflammatory factor levels in rats across each group, *n* = 3; **H** Effects of YYJH-containing serum on inflammatory factor levels (YYJH represents medicated serum), *n* = 3. Data were presented as *mean* ± *SD*. Compared to control: ^#^*p* < 0.05, ^##^*p* < 0.01, ^###^*p* < 0.001, ^####^*p* < 0.0001. Compared to model: *p < 0.05, **p < 0.01, ***p < 0.001, ****p < 0.0001

After continuous modeling with 20% ammonia solution for 3 days, statistical analysis was conducted on the body weight of each group of rats (Fig. 3A). The results showed that the body weight of the Model, YH 200 mg kg^−1^, YYJH 108 mg kg^−1^, YYJH 324 mg kg^−1^, and YYJH 972 mg kg^−1^ were significantly reduced compared with the Control (*p* < 0.05); After continuous administration for 5 days, the body weight of the YH 200 mg · kg^−1^, YYJH 108 mg kg^−1^, YYJH 324 mg kg^−1^, and YYJH 972 mg kg^−1^ significantly increased compared to the Model (*p* < 0.05).

The diet of rats in each group was recorded for 3 consecutive days of modeling (Fig. 3B). The results showed that the daily intake weight of the Control rats showed an increasing trend, while the daily intake weight of rats in the other modeling groups showed a significant decrease compared to the previous day (*p* < 0.05). From the second day of modeling, the daily intake weight of rats in the other modeling groups showed a significant decrease compared to the Control rats (*p* < 0.05).

After continuous modeling with 20% ammonia solution for 3 days, the apparent index scores of the model group were significantly higher than those of the normal group (Fig. 3C). After the completion of modeling, except for the normal group, all other groups were continuously administered for 5 days. The apparent index scores of the low-dose group, medium dose group, high-dose group, and positive drug group of Yuye Jinhua Qingre Tablets were significantly reduced compared with the model group (Fig. 3D), with the high-dose group showing the most significant downward trend.

As shown in Fig. 3E, the total number of WBC, NEU, LYM, MON, and EOS in the Model were significantly higher than those in Control (*p* < 0.05), indicating inflammation activation. After treatment with YYJH low, medium, high dose groups and positive drug group, the total WBC count, LYM, and MON were significantly reduced (*p* < 0.05); The high-dose group further significantly inhibited the increase of NEU and EOS (*p* < 0.05).

The H&E results show that the mucosal epithelium in the Control group (black arrow) is structurally intact, with no significant inflammatory cell infiltration or changes such as congestion and edema in the lamina propria (yellow arrow). The muscle layer (red arrow) exhibits a regular arrangement of muscle fibers, with a large number of salivary glands visible beneath the muscle layer. The Model group displays significant pathological changes, including mucosal necrosis and shedding, destruction of the lamina propria structure (black arrow), varying sizes of muscle cells in the muscle layer with multiple areas of mild hemorrhage (brown arrow), and mild proliferation of connective tissue in the muscle layer stroma (green arrow), along with glandular necrosis and granulocyte infiltration (red arrow). Although the YYJH 324 mg·kg^−1^ and YYJH 108 mg·kg^−1^ groups can partially alleviate inflammation, there are still pathological features such as necrosis of the mucosa and muscle cells (black arrow), infiltration of inflammatory cells (yellow and red arrows), accompanied by a considerable amount of eosinophilic exudate (blue arrow), and proliferation of connective tissue (brown arrow), as well as a reduction in glands. The YYJH 972 mg·kg^−1^ group shows significant improvement in lesions, characterized by the repair of the stratified squamous epithelium, abundant mucus glands, and no inflammatory infiltration, although there is a higher degree of local keratinization (yellow arrow). Its effects are similar to those of the positive drug group with silver-yellow granules, maintaining a relatively intact structure of the mucosa and glands, with only a small amount of glandular dilation observed. The results indicate that Yuye Jinhua Qingre tablets exhibit a dose-dependent efficacy, with high doses effectively repairing pharyngeal tissue damage and inhibiting inflammatory responses (Fig. 3F).

The levels of TNF-α and IL-6 in the serum of the model group rats were significantly increased compared to the normal group (Fig. 3G); The levels of IL-6 in the serum of rats in the low-dose group of Yuye Jinhua Qingre Tablets, the medium dose group of Yuye Jinhua Qingre Tablets, the high-dose group of Yuye Jinhua Qingre Tablets, and the positive drug Yinhuang Granules group were significantly reduced compared with the model group (*p* < 0.05); The levels of TNF-α in the serum of rats in the low-dose group of Yuye Jinhua Qingre Tablets, high-dose group of Yuye Jinhua Qingre Tablets, and positive drug Yinhuang Granules group were significantly reduced compared with the model group (*p* < 0.05).

Measure the levels of TNF-α and IL-6 in the supernatant of cell culture using an ELISA kit (Fig. 3H). The levels of IL-6 and TNF-α in the cell culture supernatant of the LPS model group were significantly increased compared to the control group (*p* < 0.05); After 24 h of intervention with drug containing serum (YYJH), the levels of IL-6 and TNF-α in the cell culture supernatant were significantly reduced compared to the LPS model group (*p* < 0.05). The above results show that the serum containing Yuye Jinhua Qingre Tablet can significantly reduce the content of inflammatory factors in LPS induced cell inflammation models, and has good in vitro anti-inflammatory effects.

### Integrating transcriptomics and proteomics reveals that YYJH can regulate genes and proteins related to anti-inflammatory responses and mucosal repair

**Fig. 4:**
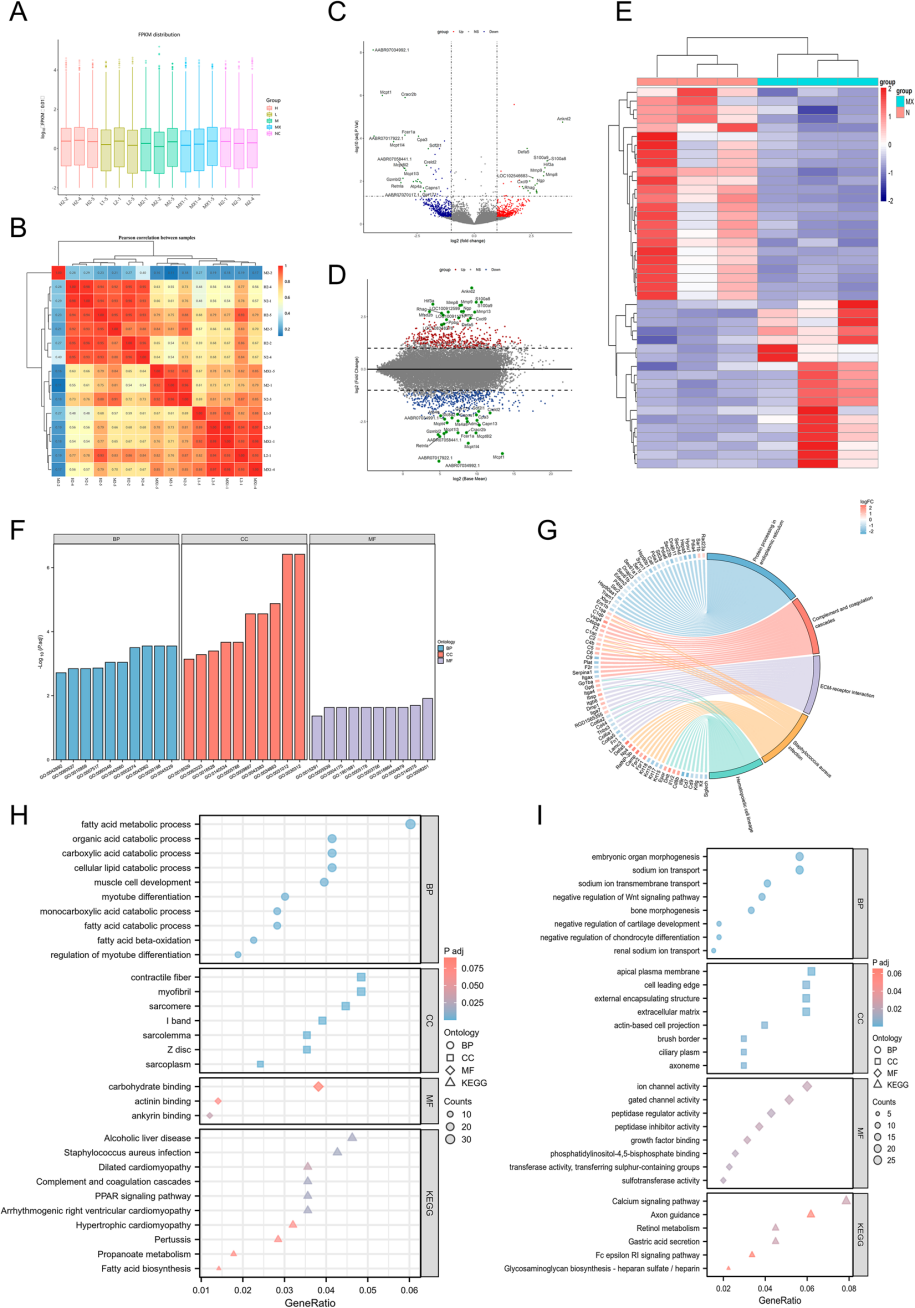
Exploring the mechanism of YYJH treatment for AP based on transcriptome sequencing. **A** Box plot of normalized raw data; **B** Heatmap of sample correlation analysis; **C** Volcano plot of differentially expressed genes between the model group and the normal group; **D** MA plot of differentially expressed genes between the model group and the normal group; **E** Heatmap of clustering of differentially expressed genes between the model group and the normal group; **F**, **G** Enrichment results of differentially expressed genes between the model group and the normal group; **H** Enrichment results of up-regulated differentially expressed genes between the model group and the normal group; **I** Enrichment results of down-regulated differentially expressed genes between the model group and the normal group. *NC* normal group, *N* normal group, *MX* model group, *L* low dose group of YYJH, *M* medium dose group of YYJH, *H* high dose group of YYJH

Compare the standardized results of various administration groups of YYJH and the correlations within the groups, along with the results of pharmacodynamic experiments, the YYJH 972 mg·kg^−1^ group was selected for subsequent transcriptomic and proteomic analyses. The results of the transcriptomic analysis showed that in the AP model group, 942 differentially expressed genes (431 upregulated and 511 downregulated) were significantly enriched in infection and inflammation-related pathways, such as the IL-17 and NOD-like receptor signaling pathways (Fig. 4), indicating that inflammatory response and immune activation are core pathological features of AP. After treatment with the YYJH 972 mg·kg^−1^ group (YH), 2317 differentially expressed genes (1404 upregulated and 913 downregulated) primarily regulated ion transport, mucosal repair, and calcium signaling pathways (Fig. S1), suggesting that it exerts anti-inflammatory effects by restoring electrolyte balance and promoting tissue regeneration.

**Fig. 5:**
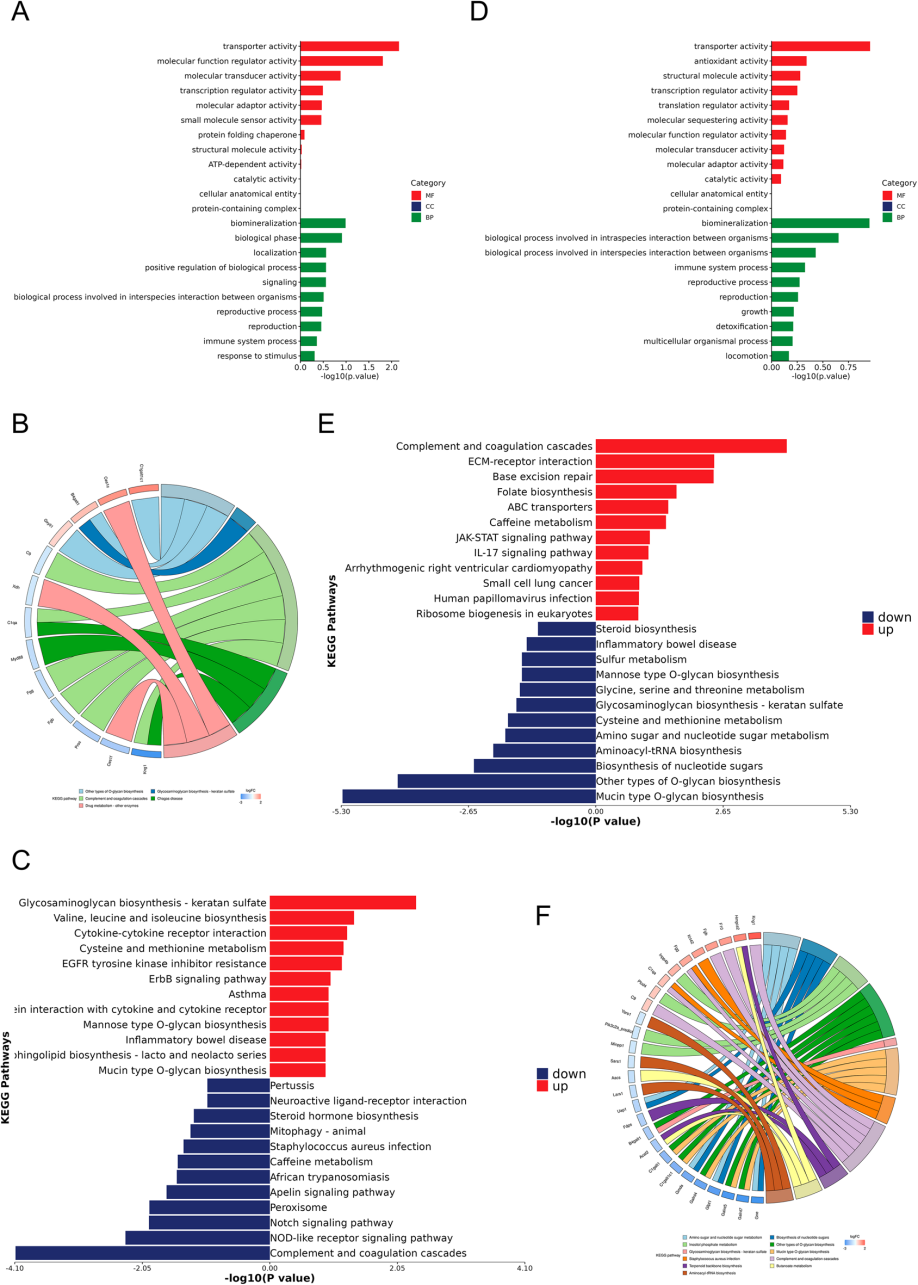
Results of GO and KEGG enrichment analysis of DEPs. **A** GO enrichment analysis results of differentially expressed proteins in acute pharyngitis; **B** KEGG enrichment analysis results of differentially expressed proteins in acute pharyngitis; **C** KEGG enrichment analysis results of up-regulated and down-regulated proteins in acute pharyngitis; **D** GO enrichment analysis results of differentially expressed proteins in acute pharyngitis treated with Yuye Jinhua Qingre Tablets; **E** KEGG enrichment analysis results of up-regulated and down-regulated proteins in acute pharyngitis treated with Yuye Jinhua Qingre Tablets; **F** KEGG enrichment analysis results of differentially expressed proteins in acute pharyngitis treated with Yuye Jinhua Qingre Tablets

Proteomics results indicate that in the AP model, 181 differentially expressed proteins (88 upregulated and 93 downregulated) are primarily involved in the complement pathway, coagulation regulation, and wound healing (Fig. S2A, B and Fig. 5A–C). In the high-dose group of Yuye Jinhua Qingre Tablet (YH) treatment, 423 differentially expressed proteins (143 upregulated and 280 downregulated) are significantly enriched in glucose metabolism, complement regulation, and nucleotide metabolism pathways (Fig. S2C, D and Fig. 5D–F), suggesting that YYJH may exert therapeutic effects by modulating the complement cascade and metabolic reprogramming.

Subcellular localization and domain analysis revealed that the differentially expressed proteins in AP were primarily localized in the nucleus and cytoplasm, being associated with the fibrinogen α/β chain family, epidermal growth factor, and PH domains. Following the high-dose group of Yuye Jinhua Qingre Tablet (YH) treatment, these differentially expressed proteins exhibited a notable shift towards cytoplasmic distribution and were enriched in both the fibrinogen α/β chain family and glycosyltransferase family 2 domains (Fig. S3).

### YYJH balances immune-metabolic dysregulation through the key molecule C9 and promotes the mucosal repair of acute pharyngitis.

**Fig. 6:**
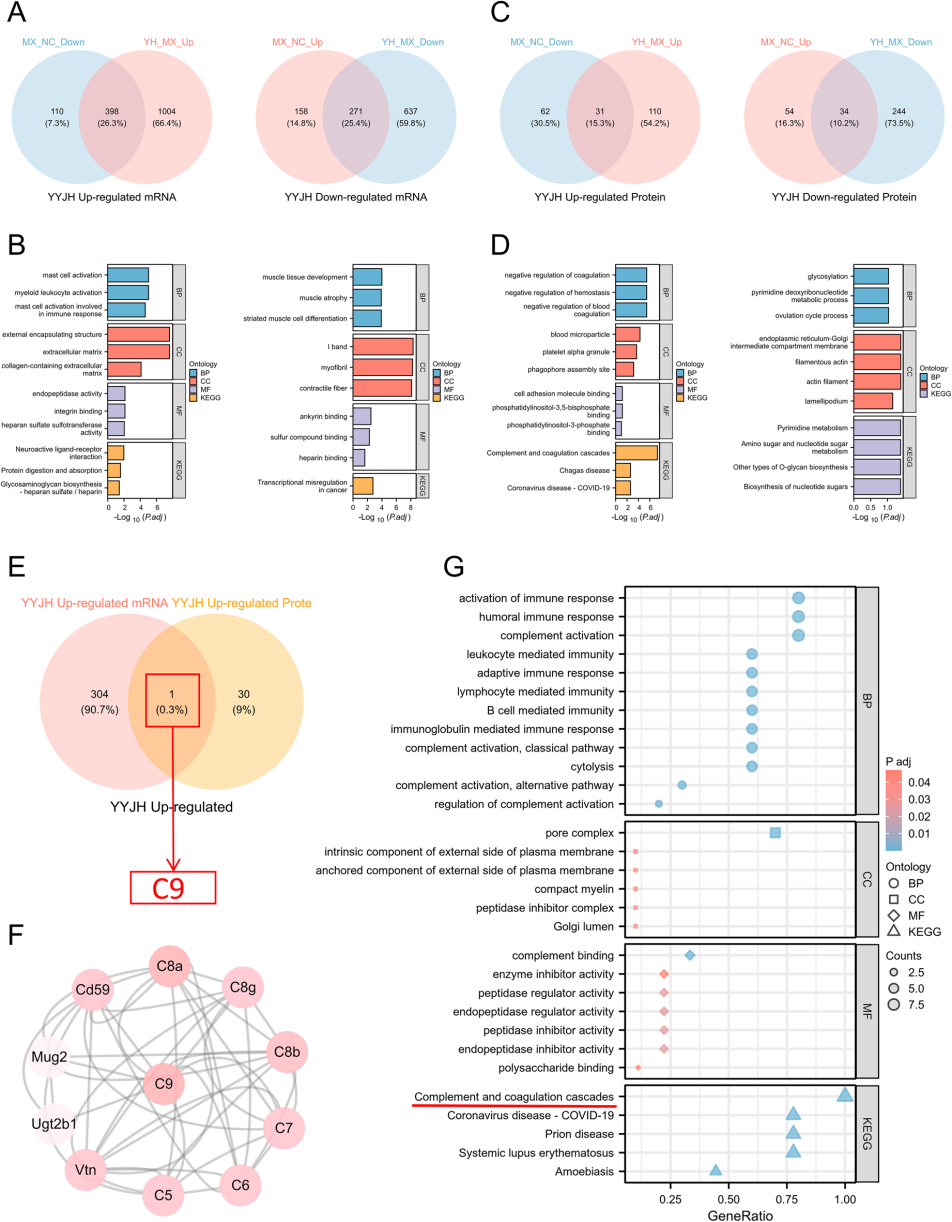
Key molecular screening and functional pathway enrichment analysis results of YYJH regulation of AP abnormal expression. **A** Venn diagram of YYJH-regulated AP abnormally expressed gene screening; **B** Bar chart of functional pathway enrichment for YYJH-regulated AP abnormally up-regulated and down-regulated genes; **C** Venn diagram of YYJH-regulated AP abnormally expressed protein screening; **D** Bar chart of functional pathway enrichment for YYJH-regulated AP abnormally up-regulated and down-regulated proteins; **E** Venn diagram of YYJH-regulated AP abnormally expressed key molecule screening; **F** PPI network diagram of key molecules; **G** Bubble chart of functional and pathway enrichment for key molecules. *NC* normal group, *MX* model group, *H* high dose group of YYJH

YYJH upregulated 398 abnormal expression genes in acute pharyngitis(Fig. 6A), which are associated with immune cell activation and inflammation regulation, mucosal barrier repair and extracellular matrix remodeling, tissue repair and secretion regulation, enzyme activity and metabolic support, as well as neuro-immune-mucosal interactions (Fig. 6B). A total of 271 downregulated genes (Fig. 6A) are related to the regulation of ion homeostasis and muscle contraction function, maintenance of muscle structural stability, alleviation of oxidative stress and toxic stimuli, and regulation of the local microenvironment and signal transduction (Fig. 6B).

YYJH upregulated 31 abnormal expression proteins in acute pharyngitis (Fig. 6C), which are linked to anticoagulation and microcirculation regulation, immune inflammation and complement system regulation, tissue repair and fibrosis inhibition, pathogen defense and immune evasion intervention, as well as cell migration and barrier function maintenance (Fig. 6D). Conversely, 34 downregulated proteins (Fig. 6C) are associated with the regulation of glycosylation modifications and protein secretion, regulation of cytoskeletal dynamics and migration, metabolic reprogramming, and energy balance (Fig. 6D).

YYJH regulates the key molecule complement component 9 (C9) in acute pharyngitis, which is primarily enriched in pathways related to the complement pathway, systemic lupus erythematosus, prion disease, coronavirus disease—COVID-19, and amoebiasis (Fig. 6E–G).

**Fig. 7:**
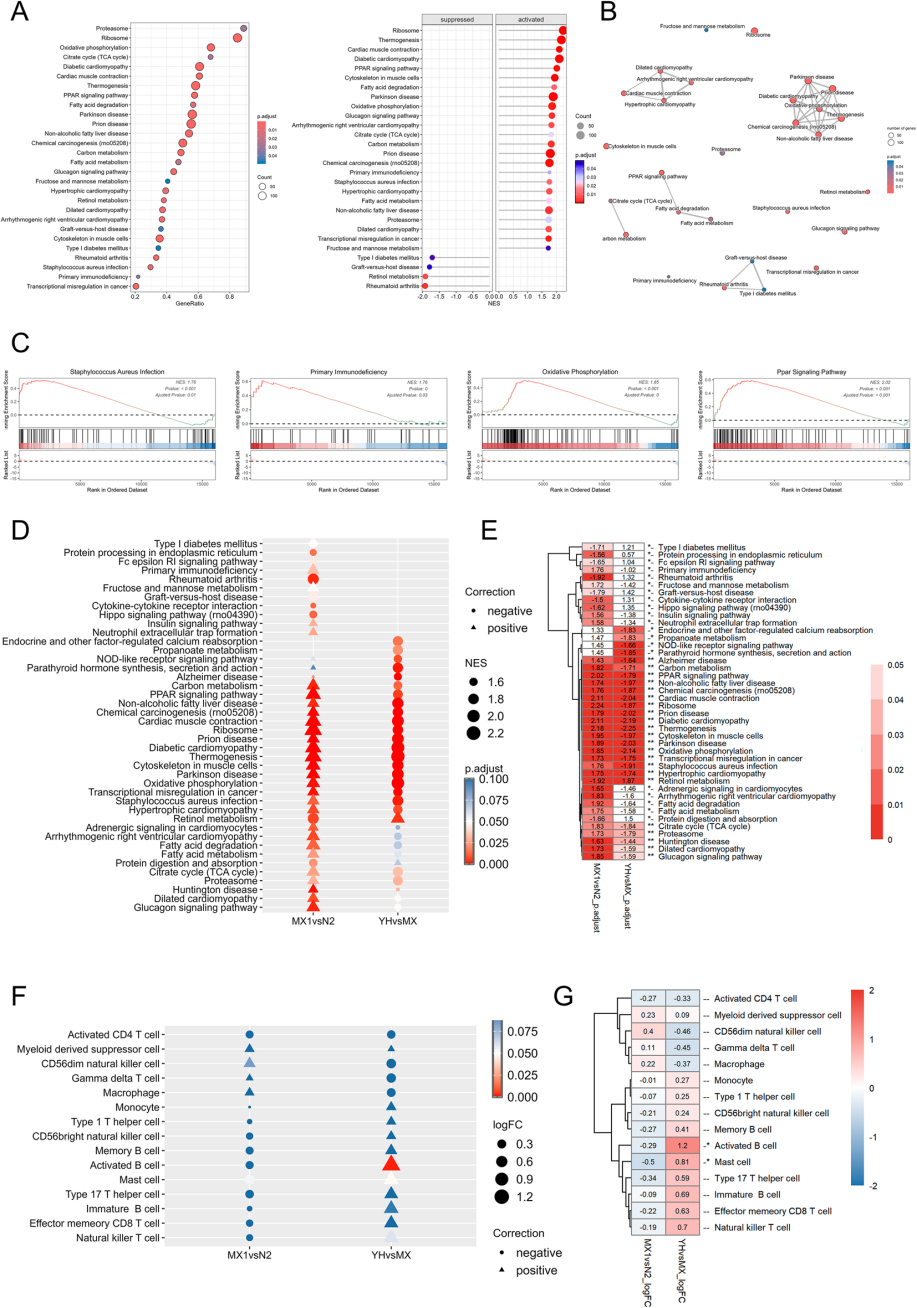
Gene set enrichment analysis results. **A** Bubble chart of enriched abnormally expressed genes in AP; **B** Enrichment network diagram; **C** Enrichment GSEA plot; **D** Bubble chart of gene set differences between abnormally expressed genes in AP and YYJH-treated abnormally expressed genes in AP; **E** Heatmap of gene set differences; **F** Bubble chart of enrichment differences in gene sets between abnormally expressed genes in AP and YYJH-treated abnormally expressed genes in AP; **G** Heatmap of gene set differences. *N2* normal group, *MX* model group, *MX1* model group, *H* high dose group of YYJH

Gene set enrichment analysis indicates that the PPAR signaling pathway, oxidative phosphorylation, and Staphylococcus aureus infection pathways are significantly activated among the differentially expressed genes in acute pharyngitis (Fig. 7A–C), whereas these pathways are significantly suppressed in the differentially expressed genes treated with YYJH for acute pharyngitis (Fig. S4). GSVA analysis results show that gene sets related to the PPAR signaling pathway, oxidative phosphorylation, and Staphylococcus aureus infection are significantly activated in the differentially expressed genes of acute pharyngitis and significantly suppressed in the differentially expressed genes treated with YYJH, revealing an opposing trend (Fig. 7D, E). Further analysis of cell states among the groups reveals that macrophages exhibit an activated state in the differentially expressed genes of acute pharyngitis, while they present a suppressed state in the differentially expressed genes treated with YYJH for acute pharyngitis (Fig. 7F, G), suggesting that YYJH can reverse the abnormal activation of immune cells in acute pharyngitis.

### The anti-inflammatory mechanism of Yuye Jinhua Qingre Tablets by inhibiting the activation of complement C5a/C5aR1 axis

**Fig. 8:**
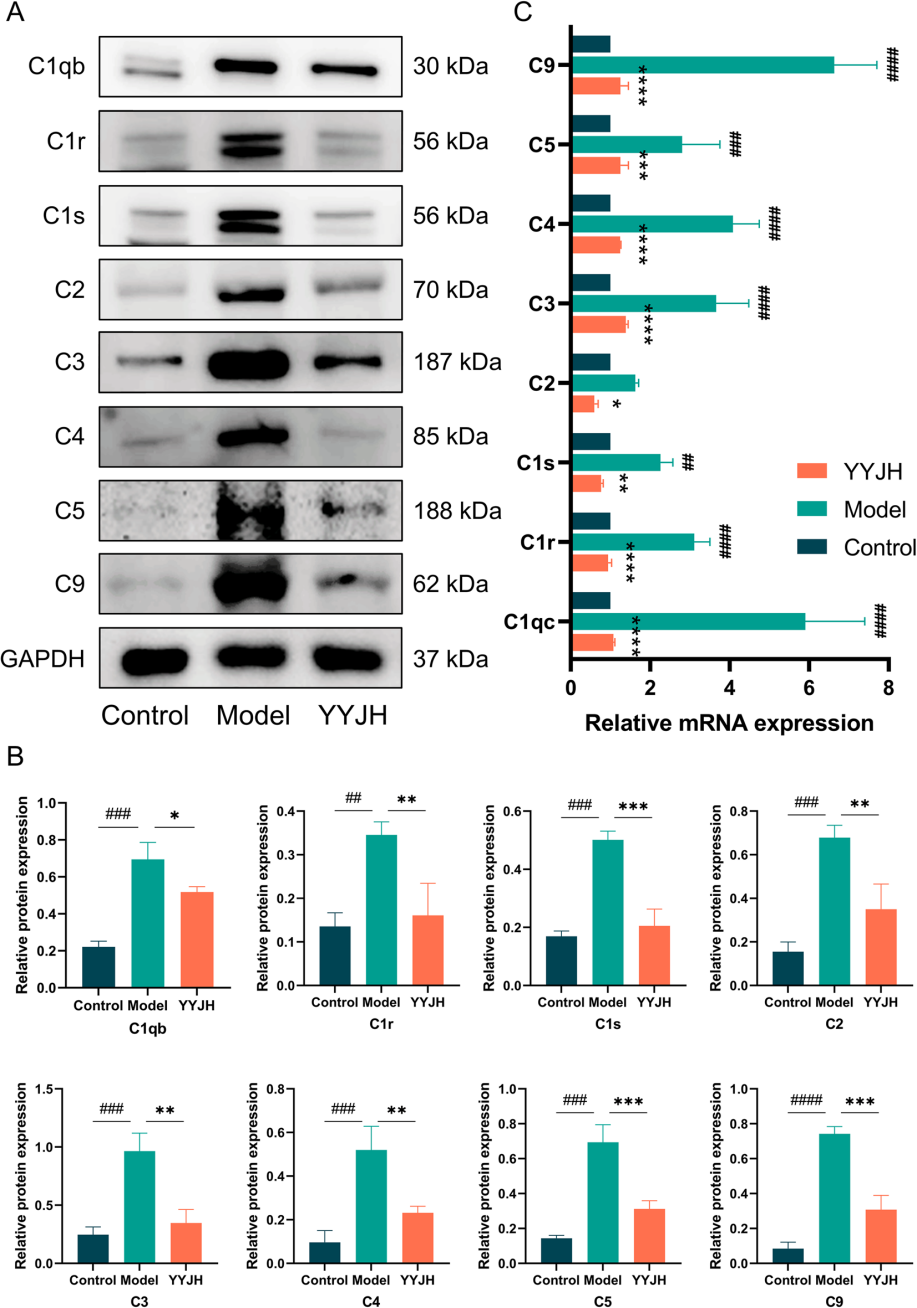
YYJH inhibits activation of the classical complement pathway. **A** Western blot results of complement pathway-related proteins; **B** Statistical results of expression levels of complement pathway-related proteins; **C** RT-qPCR experimental results. Data were presented as *mean* ± *SD*, *n* = 3. Compared to control: ^##^*p* < 0.01, ^###^*p* < 0.001, ^####^*p* < 0.0001. Compared to model: *p < 0.05, **p < 0.01, ***p < 0.001, ****p < 0.0001

Compared with the Control group, the expression level of complement proteins in the Model group was significantly upregulated (Fig. 8), specifically manifested as upregulation of key components of the classical complement pathway, C1 complex (C1r, C1s, C1qb), and downstream Complement protein 2 (C2) and Complement protein 4 (C4) expression; The synchronous upregulation of Complement protein 3 (C3), Complement protein 5 (C5), and membrane attack complex component C9 in the complement common terminal pathway suggests the comprehensive activation of the complement system from classical pathways to terminal effects. Compared with the Model group, the expression of complement protein C1 complexes (C1r, C1s, C1qb) and downstream C2, C4, as well as common terminal pathway components C3, C5, and C9, were significantly reduced in the YYJH group (*p* < 0.05). The above results suggest that Yuye Jinhua Qingre Tablets may inhibit the classical activation pathway of complement, downregulate the expression of C1 complex and C2, C4, and block the initiation of classical pathways; Simultaneously regulating the complement cascade amplification effect, reducing the expression levels of C3, C5, and C9, and decreasing the formation of membrane attack complexes.

**Fig. 9:**
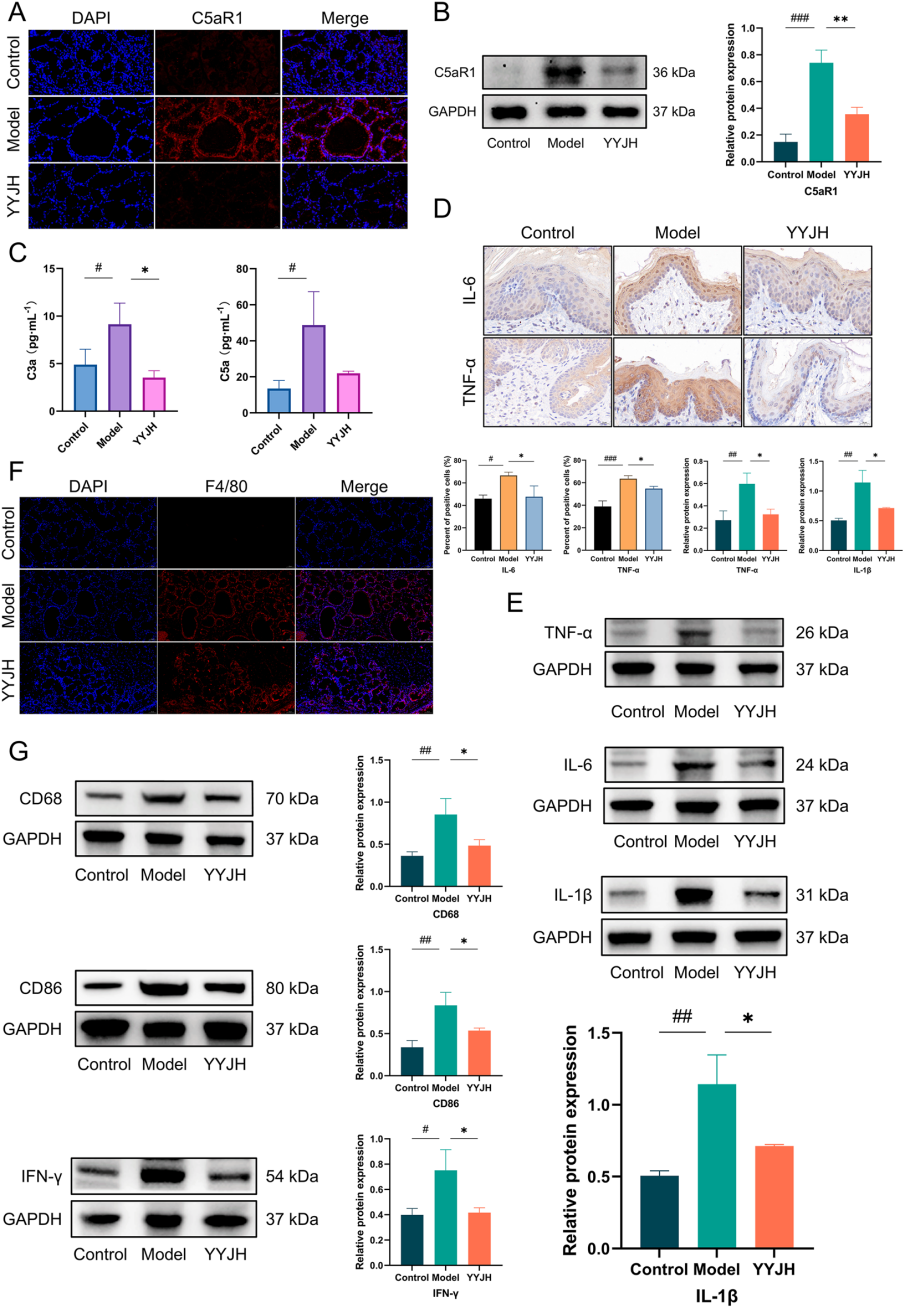
YYJH inhibits the activation of complement C5a/C5aR1 axis to exert anti-inflammatory effects. **A** IF experimental results of YYJH intervention on the expression of molecules related to the complement pathway (bar = 20 μm); **B** Western blot experimental results of YYJH intervention on the expression levels of molecules related to the complement pathway; **C** ELISA detection results of YYJH intervention on the expression levels of molecules related to the complement pathway; **D** IHC experimental results of YYJH regulation on the levels of inflammatory factors (bar = 20 μm); **E** Western blot experimental results of YYJH regulation on the expression levels of inflammatory factors; **F** IF experimental results of YYJH regulation on the expression of macrophage markers (bar = 50 μm); **G** Western blot experimental results of YYJH regulation on the expression levels of molecules related to macrophage activation. Data were presented as *mean* ± *SD*, *n* = 3. Compared to control: ^#^*p* < 0.05, ^##^*p* < 0.01, ^###^*p* < 0.001. Compared to model: *p < 0.05, **p < 0.01

To elucidate the molecular mechanism of YYJH in regulating the complement pathway, the expression levels of the inflammatory mediators C5a and C3a downstream of the classical activation pathway of complement were detected by ELISA (Fig. 9C). The ELISA experiment results showed that compared with the Control group, the expression levels of C5a and C3a in the Model group were significantly upregulated (*p* < 0.05); Compared with the Model group, the expression levels of C5a and C3a were downregulated in the YYJH group. The activation of the complement system leads to the release of inflammatory mediators. YYJH can inhibit the complement cascade reaction, reduce the production of C5a and C3a, and decrease the release of inflammatory mediators (Fig. 9E).

The Western blot experiment results showed that compared with the Control group, the expression level of C5aR1 protein in the Model group was significantly upregulated (*p* < 0.05), while after intervention with YYJH, its expression level was significantly reduced compared with the Model group (*p* < 0.05) (Fig. 9B). IF experiments further confirmed that the fluorescence intensity of C5aR1 on the surface of the cell membrane in the Model group was significantly enhanced compared to the Control group, while the membrane localization signal in the YYJH group was significantly weakened compared to the Model group (Fig. 9A). It was shown that YYJH can downregulate the expression of C5aR1, and inhibit the membrane localization signal of C5aR1, thereby blocking G protein coupled signal transduction.

Further detection of macrophage phenotype polarization and expression characteristics of inflammatory factors. IHC analysis showed that the protein expression levels of pro-inflammatory cytokines IL-6 and TNF-α in the pharyngeal mucosal tissue of the Model group were significantly increased compared to the Control group (*p* < 0.05), while their expression was significantly inhibited after intervention with YYJH (*p* < 0.05) (Fig. 9D), indicating that the drug can effectively block the release of local inflammatory mediators. Further analysis by IF and Western blot (Fig. 9F, G) revealed that the expression of macrophage markers F4/80, CD68, and M1 polarization marker CD86 was significantly upregulated in the Model group (*p* < 0.05), while the secretion levels of pro-inflammatory factors IFN-γ, IL-1β, TNF-α, and IL-6 increased synchronously (*p* < 0.01). After treatment with YYJH, the expression of the above molecules was significantly reduced (*p* < 0.05), indicating that the drug may inhibits the C5a/C5aR1 axis activity, blocks macrophage polarization towards the pro-inflammatory phenotype (M1), and thereby inhibits the inflammatory cascade reaction.

## Discussion

Acute pharyngitis (AP), a highly prevalent infectious disease of the upper respiratory tract, involves a pathogenic mechanism that encompasses various factors, including bacterial and viral infections as well as physical and chemical irritations [1]. Modern medicine primarily employs glucocorticoids and broad-spectrum antibiotics for treatment; however, long-term use can lead to side effects such as microbial dysbiosis and immune suppression [13–15]. Traditional Chinese Medicine (TCM) categorizes this condition under “throat obstruction” and advocates for multi-target regulation through compound herbal medicines that clear heat, detoxify, diffuse the lungs, and dispel phlegm. Among these, YYJH and other compound preparations have garnered attention due to their multi-component synergistic effects [16–18]. However, analyzing the pharmacodynamic material basis of TCM compounds presents a core challenge: traditional research has primarily focused on the components of medicinal materials, while the blood-entering components (including prototype compounds and their metabolites) that actively participate in target regulation have not been systematically elucidated. This lack of clarity complicates the establishment of the correlation mechanism between in vitro active components and the overall therapeutic efficacy in vivo [19]. With the advancement of systems biology technologies, multi-dimensional omics approaches, such as transcriptomics and proteomics, have provided new paradigms for revealing the 'multi-component, multi-pathway, multi-target' regulatory network of TCM. The high compatibility of these approaches with the holistic view of TCM has been validated in antiviral studies of compounds such as Lianhua Qingwen Capsule [20, 21].

The complement system, serving as the core effector mechanism of innate immunity, is intricately linked to the pathological processes of inflammatory diseases due to its abnormal activation [22–24]. The classical pathway of complement is initiated by the recognition of antigen–antibody complexes mediated by C1q, which triggers the cleavage cascade of C4/C2, resulting in the generation of C3 convertase (C4b2a) and the membrane attack complex (MAC). These components directly participate in inflammatory responses, such as mucosal congestion in the pharynx and hyperplasia of lymphoid follicles [25]. Notably, the overactivation of the C5a/C5aR1 signaling axis can induce a cytokine storm characterized by the release of IL-6 and TNF-α through the activation of the MAPK/NF-κB pathway. This process also promotes the formation of neutrophil extracellular traps (NETs), exacerbating microcirculatory disturbances and multi-organ damage [26–28]. Although anti-C5a monoclonal antibodies have demonstrated efficacy in severe conditions such as sepsis and ARDS, their single-target nature and comprehensive inhibition of the upstream complement pathway may pose infection risks. This has prompted the academic community to shift its focus towards multi-target regulatory strategies utilizing natural medicines [29, 30]. Yuye Jinhua Qingre Tablets contain blood-entering components, including geniposide and andrographolide, and literature suggests that they may intervene in the complement cascade by inhibiting C5aR1 activity. However, it remains to be experimentally verified whether this intervention occurs through the regulation of the C1q-C4b2a-MAC pathway or the blockade of the C5a/C5aR1 axis signaling [31, 32].

This study systematically elucidates the pharmacodynamic mechanisms and molecular regulatory networks of YYJH in treating AP through a multi-dimensional integrated analysis. At the pharmacodynamic validation level, twelve active components that entered the bloodstream were identified using UPLC-Q-Exactive-Orbitrap-MS technology, confirming that the drug significantly alleviates clinical symptoms of AP in rats. The high-dose group effectively restored the body weight of the model animals, while all dosage groups exhibited anti-inflammatory effects by inhibiting pharyngeal mucosal cell necrosis, reducing inflammatory cell infiltration, decreasing peripheral blood inflammatory cell counts, and lowering serum levels of inflammatory factors such as IL-6 and TNF-α. Furthermore, the LPS-induced NP69SV40T cell model in vitro further validated that the drug-containing serum could inhibit the release of inflammatory mediators, indicating the consistency of its anti-inflammatory effects both in vivo and in vitro.

**Fig. 10:**
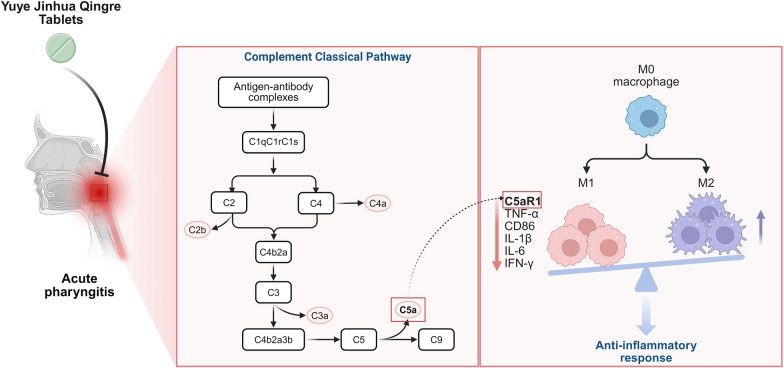
YYJH inhibits the activation of complement C5a/C5aR1 axis to exert anti-inflammatory effects

At the mechanistic analysis level, the integration of transcriptomics and proteomics cross-analysis revealed that the drug primarily reverses the metabolic-immune imbalance in the AP model by regulating biological processes such as antibacterial humoral immunity and the IL-17 signaling pathway. Gene Set Enrichment Analysis (GSEA) indicated that the characteristic gene sets abnormally activated in the model group, including carbon metabolism, oxidative phosphorylation, the PPAR signaling pathway, and dendritic cell/macrophage activation, exhibited a significant inhibitory trend in the drug-administered group. Through an in-depth analysis of omics data, the key molecule C9 in the complement pathway was identified as the core regulatory target, establishing a pathological mechanism framework of "Staphylococcus aureus infection—complement cascade activation—C5a/C5aR1 axis-mediated inflammatory response." Experimental results demonstrated that YYJH effectively blocks the activation of the C5a/C5aR1 signaling axis by downregulating C5aR1 protein expression, inhibiting C5 cleavage, and reducing macrophage infiltration. Consequently, it regulates the downstream inflammatory cytokine network, including IL-6 and TNF-α, thereby forming a comprehensive regulatory pathway of "pathogen recognition—complement activation—inflammatory cascade." The graphical abstract of this study is shown in Fig. 10.

## Conclusions

This study adopts a research approach that integrates pharmacodynamic experiments, transcriptomics, and proteomics with drug mechanism validation to elucidate how YYJH exerts a synergistic anti-inflammatory and repair effect by inhibiting the complement cascade (C2–C5–C9), blocking C5a/C5aR1 signaling, and regulating macrophage phenotype transformation. This research provides a theoretical basis for a scientific explanation of the anti-inflammatory effects of YYJH and lays the groundwork for its precise clinical application and further in-depth studies post-market.

## Supplementary Information

Additional file 1: Fig. S1 Exploring the mechanism of YYJH treatment for AP based on transcriptome sequencing. Fig. S2 Analysis results of DEPs. Fig. S3 Results of subcellular localization and structural domain analysis of DEPs. Fig. S4 Gene set enrichment analysis results.

Additional file 2.

Additional file 3.

## Data Availability

Data will be made available on request.

## Abbreviations

BP: Biological process
BSA: Bovine serum albumin
CC: Cellular component
CCK-8: Cell counting kit-8
DEGs: Differentially expressed genes
DEPs: Differentially expressed proteins
ELISA: Enzyme-linked immunosorbent assay
ESI: Electrospray source ionization
GO: Gene ontology
IHC: Immunohistochemistry
KEGG: Kyoto Encyclopedia of Genes and Genomes
NEU: Number of neutrophil
OD: Optical density
PFA: Paraformaldehyde
PPI: Protein–protein interactions
qPCR: Quantitative polymerase chain reaction
RBC: Red blood cell
RT-qPCR: Reverse transcription quantitative polymerase chain reaction
TCM: Traditional Chinese medicine
TMT: Tandem mass tag
WBC: White blood cell
