# Supplementary material for: Yuye Jinhua Qingre Tablets Attenuate Acute Pharyngitis by inhibiting the Complement Cascade and C5a/C5aR1 Axis
Source: Chin Med. 2025 Aug 25;20:134. doi: 10.1186/s13020-025-01191-1 (PMC12376329; doi:10.1186/s13020-025-01191-1)
Supplement: Supplementary file 1 — Additional file 1: Fig. S1 Exploring the mechanism of YYJH treatment for AP based on transcriptome sequencing. Fig. S2 Analysis results of DEPs. Fig. S3 Results of subcellular localization and structural domain analysis of DEPs. Fig. S4 Gene set enrichment analysis results. [file 13020_2025_1191_MOESM1_ESM.docx]

# **Supplementary Figures.**


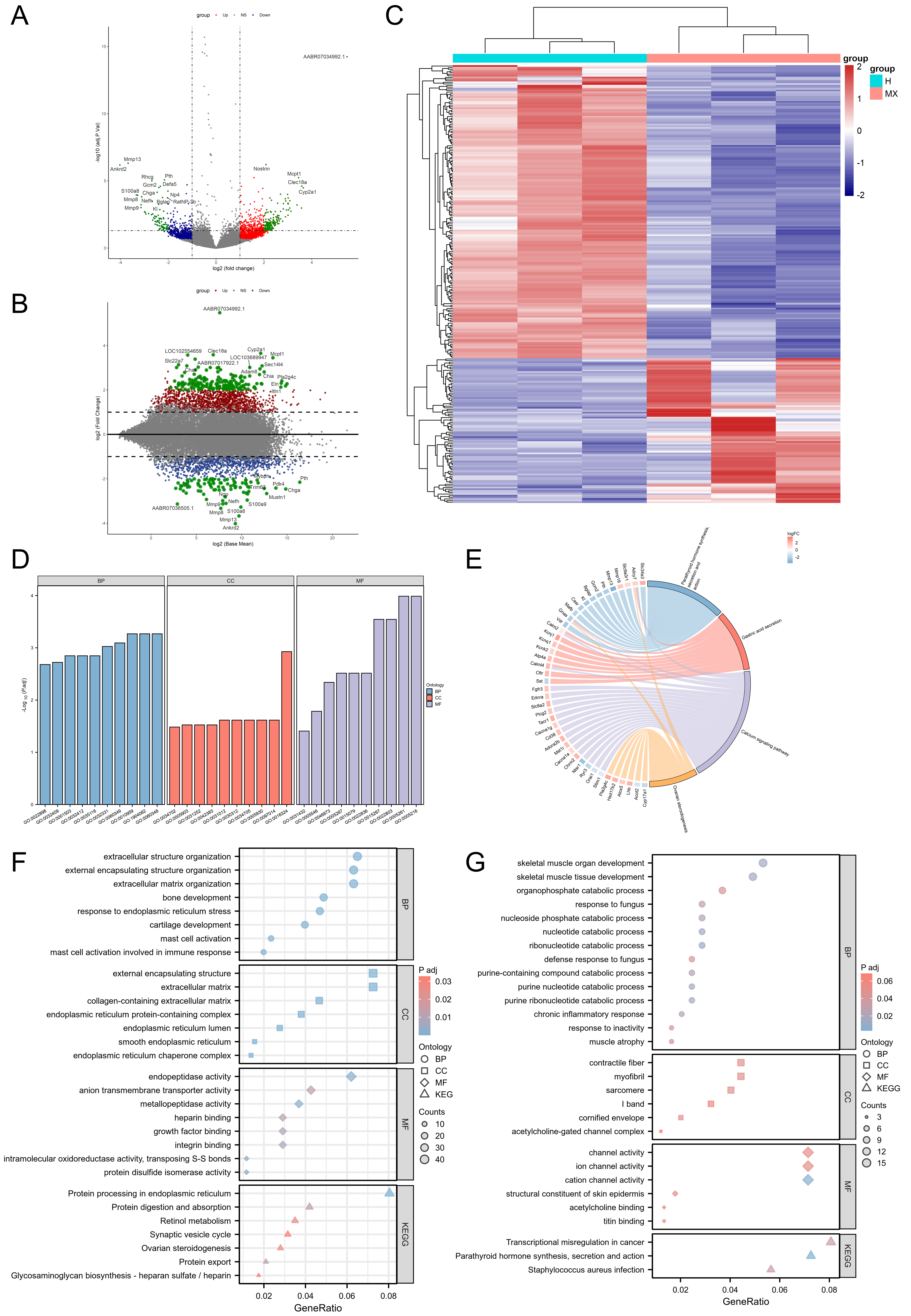


**Fig. S1** Exploring the mechanism of YYJH treatment for AP based on transcriptome sequencing.

**A** Volcano plot of differentially expressed genes between the YYJH group and the model group; **B** MA plot of differentially expressed genes between the YYJH group and the model group; **C** Heatmap of clustering of differentially expressed genes between the YYJH group and the model group; **D-E** Enrichment results of differentially expressed genes between the YYJH group and the model group; **F** Enrichment results of up-regulated differentially expressed genes between the YYJH group and the model group; **G** Enrichment results of down-regulated differentially expressed genes between the YYJH group and the model group.


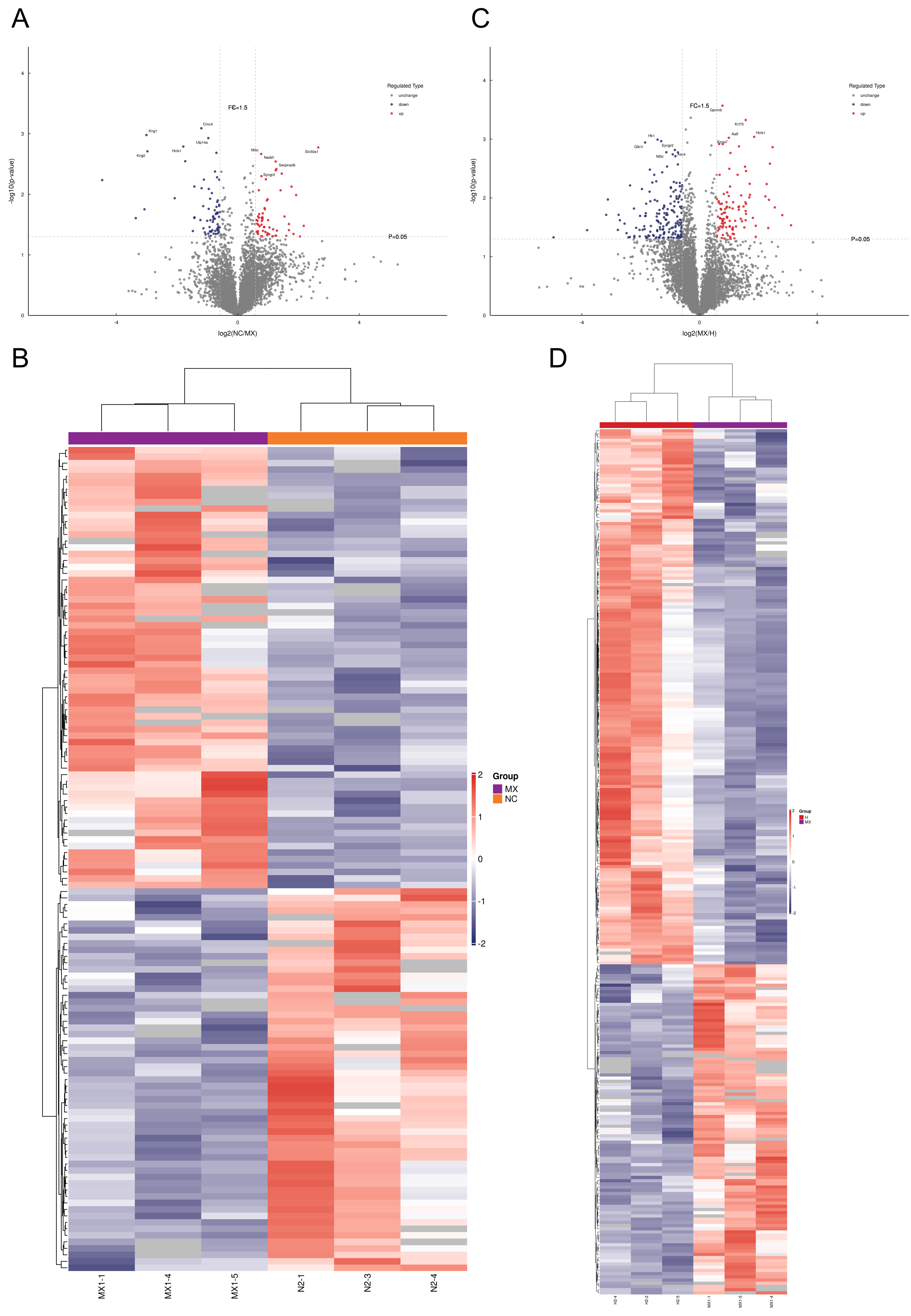


**Fig. S2** Analysis results of DEPs.

**A-B** Volcano plots and clustering heatmaps of differentially expressed proteins between the model group and the normal group; **C-D** Volcano plots and clustering heatmaps of differentially expressed proteins between the treatment group and the model group. (NC: Normal group; MX: Model Group; H: High dose group of YYJH)

**
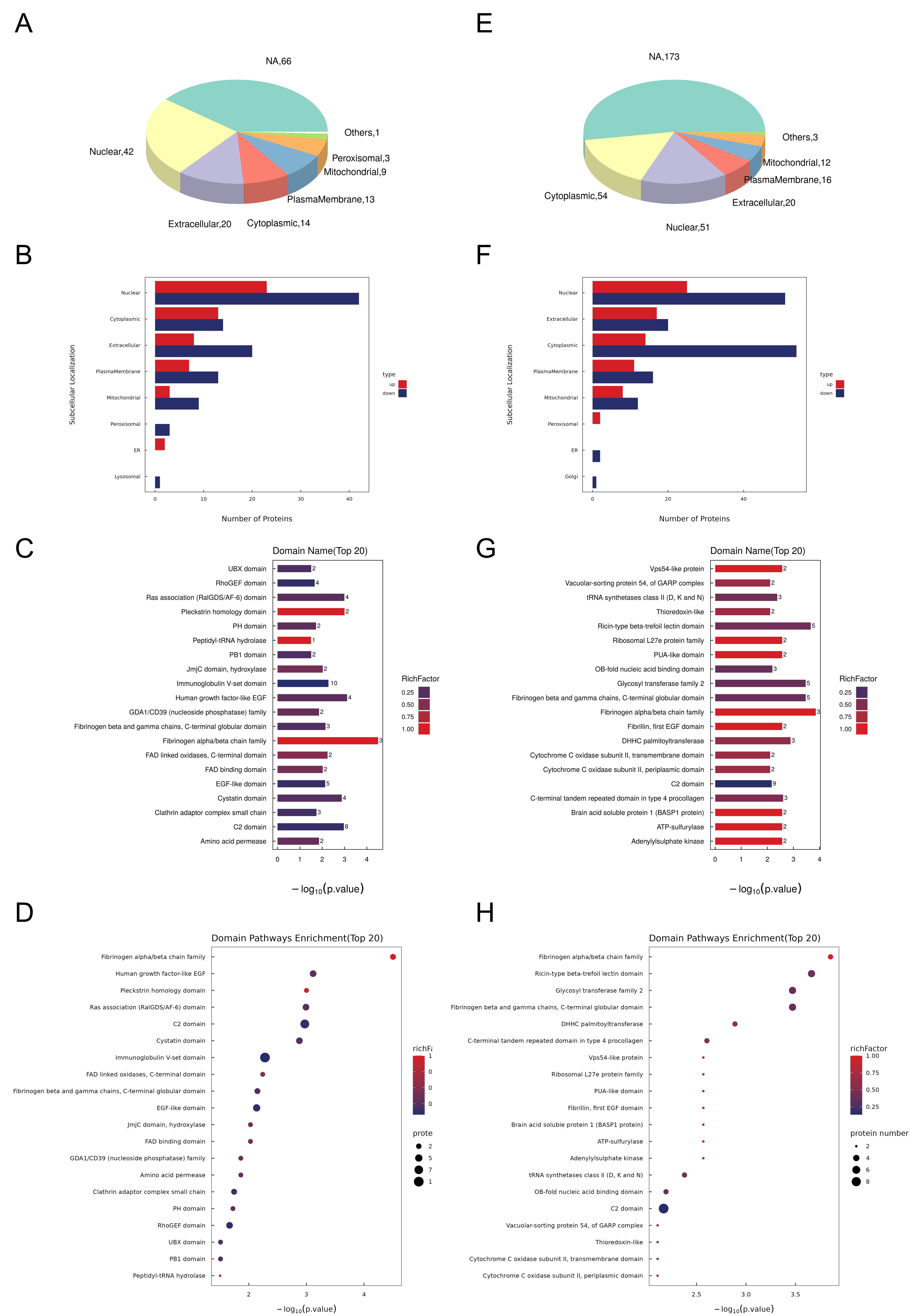
**

**Fig. S3** Results of subcellular localization and structural domain analysis of DEPs.

**A** Pie chart illustrating the distribution of subcellular organelle localization of differentially expressed proteins between the model group and the normal group; **B** Bar chart depicting the subcellular organelle localization of differentially expressed proteins between the model group and the normal group; **C** Bar chart representing the domain analysis of differentially expressed proteins between the model group and the normal group; **D** Bubble chart showing the domain enrichment of differentially expressed proteins between the model group and the normal group; **E** Pie chart illustrating the distribution of subcellular organelle localization of differentially expressed proteins between the administration group and the model group; **F** Bar chart depicting the subcellular organelle localization of differentially expressed proteins between the administration group and the model group; **G** Bar chart representing the domain analysis of differentially expressed proteins between the administration group and the model group; **H** Bubble chart showing the domain enrichment of differentially expressed proteins between the administration group and the model group.


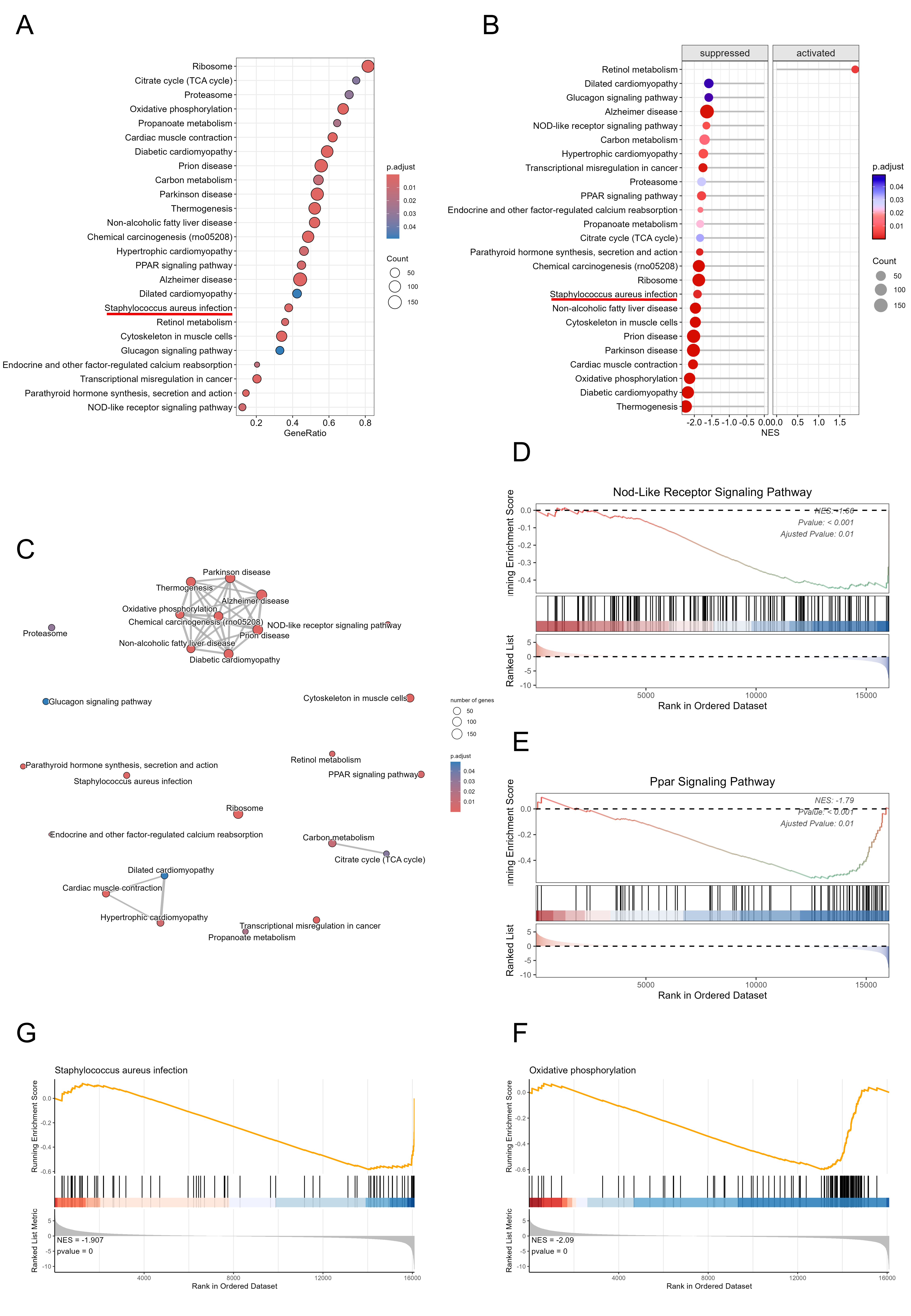


**Fig. S4** Gene set enrichment analysis results.

**A-B** Bubble chart of enriched abnormally expressed genes in YYJH regulated AP; **C** Enrichment network diagram; **D** Enrichment GSEA plot.

# **Supplementary Methods.**

**RT-qPCR analysis**

The target gene primer sequences used are as follows:

Rat-C9-Forward: CAAACAAAGGTTTCGCTCAAGAA

Rat-C9-Reverse: ACAGTCGTTGTCACCATTACAC

Rat-C1qc-Forward: AGAAGCACCAGTCGGTATTCA

Rat-C1qc-Reverse: TGCGATGTGTAGTAGACGAAGTA

Rat-C1r-Forward: TTTGGGGAATCCTCCATACTTGC

Rat-C1r-Reverse: GTGACAGAGTCGCCTTCACAG

Rat-C1s-Forward: GCTGCTCACGTTTTGGAGAAA

Rat-C1s-Reverse: AGCTGGGATGAATAAAGACACG

Rat-C2-Forward: CAGAGCAATGGAGTGTGGAGTGG

Rat-C2-Reverse: GTTGGTGAGGGAGGTGTCTAGGG

Rat-C4-Forward: TTGACAGTGCGGCTTCCTGATTC

Rat-C4-Reverse: AACACGAGTTGGCTTGGCTACAC

Rat-C3-Forward: ACTGTGGACAACAACCTACTGC

Rat-C3-Reverse: GCATGTTCGTAAAAGGCTCGG

Rat-C5-Forward: TACATAGTCACGGGGGAGCA

Rat-C5-Reverse: ACTCGTTGAAGGCCCTGATG

Rat-Gapdh-Forward: AGGTCGGTGTGAACGGATTTG

Rat-Gapdh-Reverse : GGGGTCGTTGATGGCAACA
