# Supplementary material for: Yuye Jinhua Qingre Tablets Attenuate Acute Pharyngitis by inhibiting the Complement Cascade and C5a/C5aR1 Axis
Source: Chin Med. 2025 Aug 25;20:134. doi: 10.1186/s13020-025-01191-1 (PMC12376329; doi:10.1186/s13020-025-01191-1)

c1qb

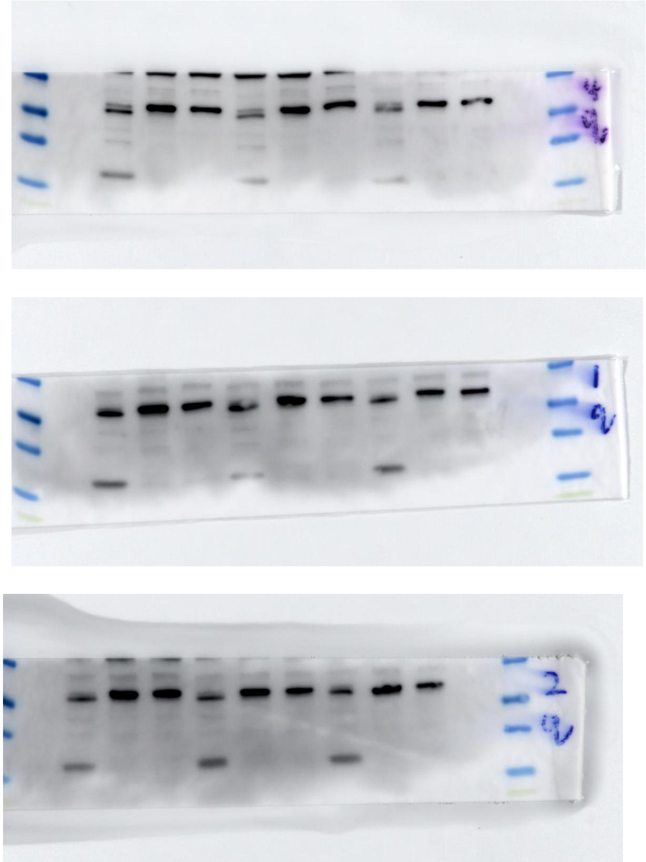

gapdh

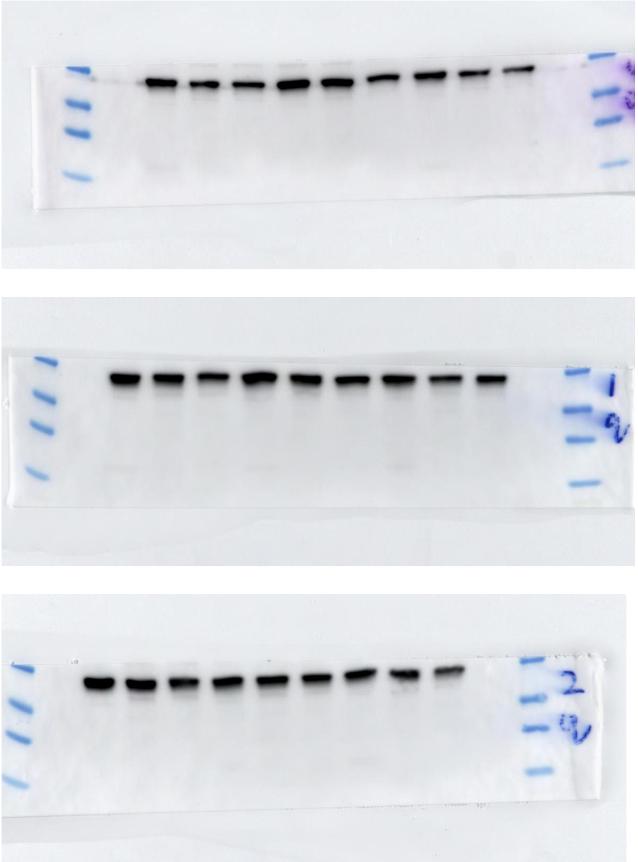

c1r

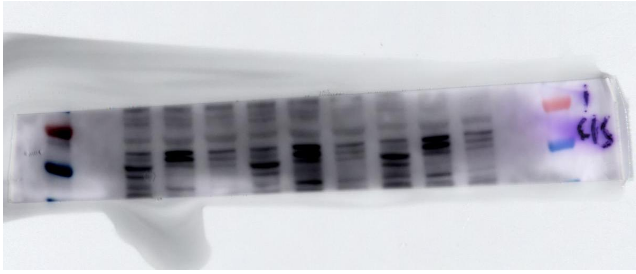

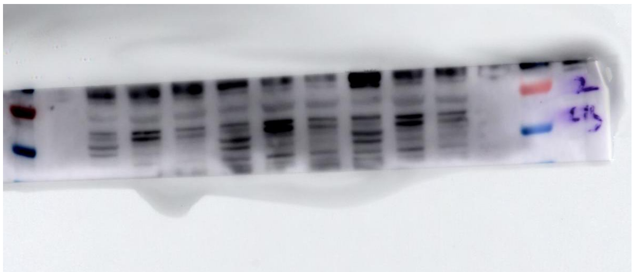

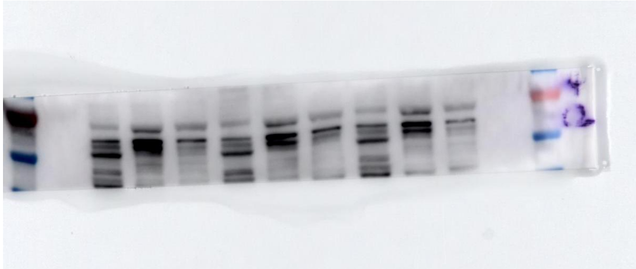

gapdh

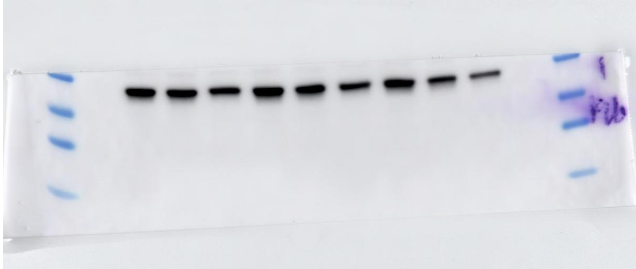

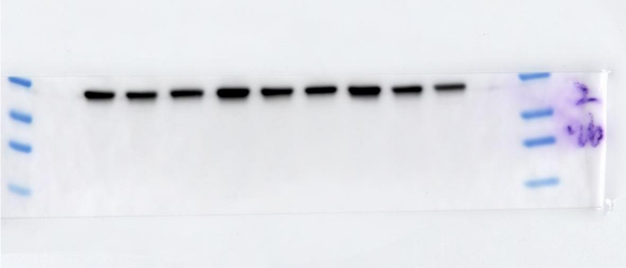

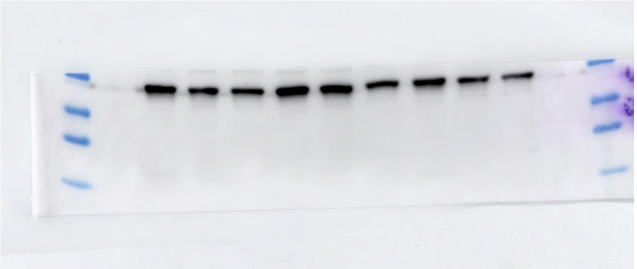

c1s

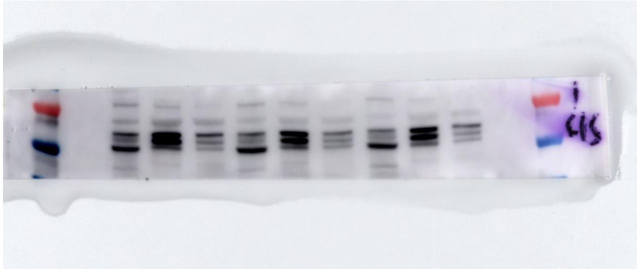

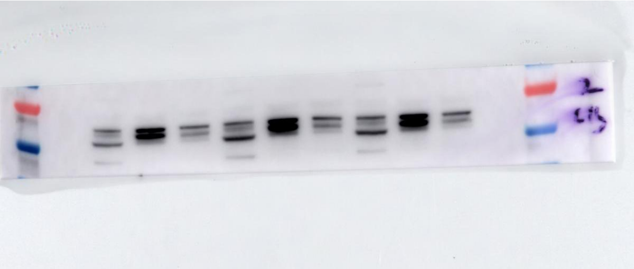

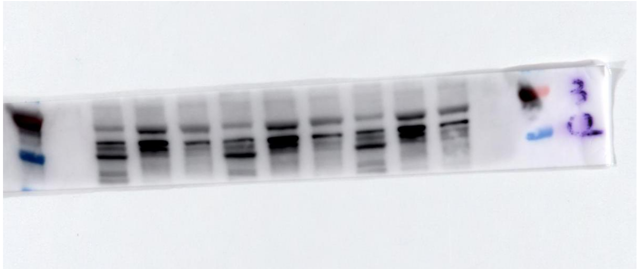

gapdh

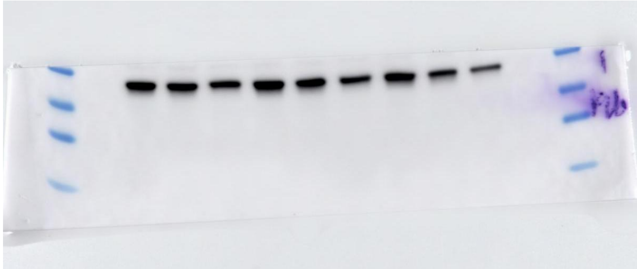

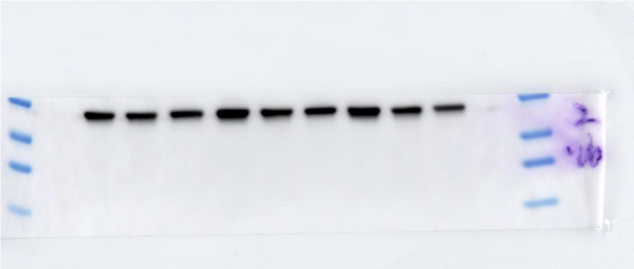

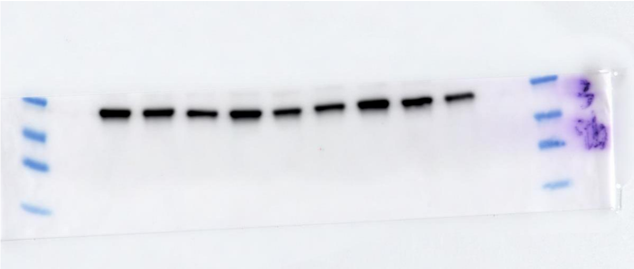

c2

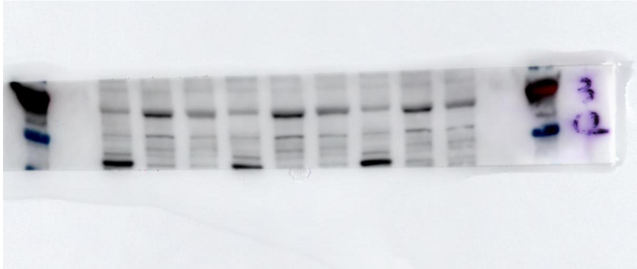

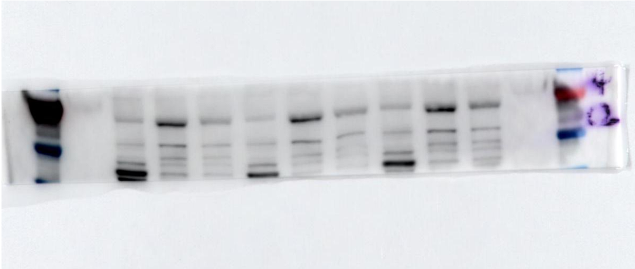

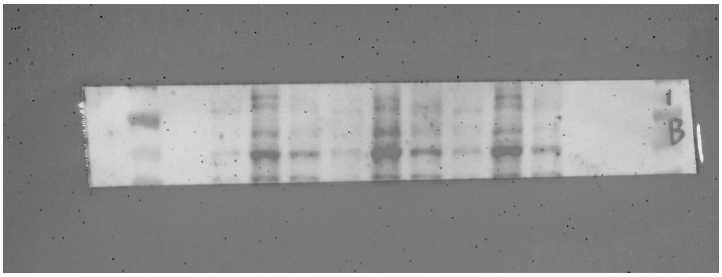

gapdh

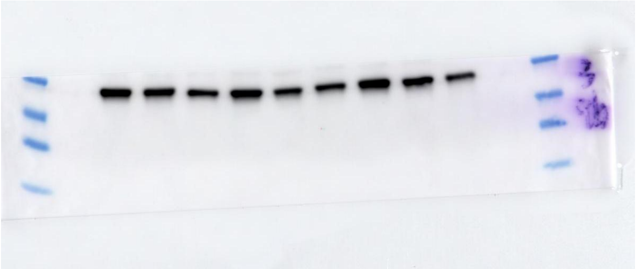

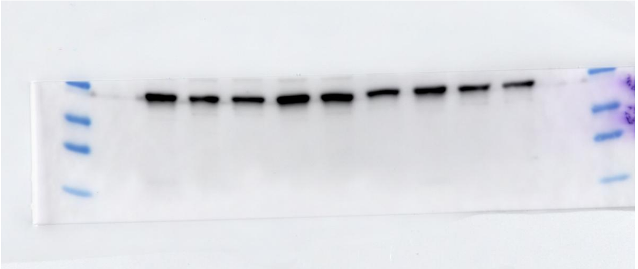

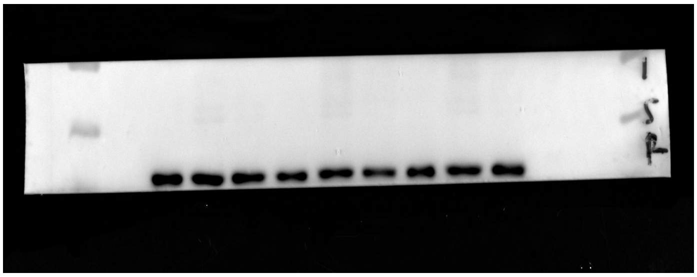

c3

gapdh

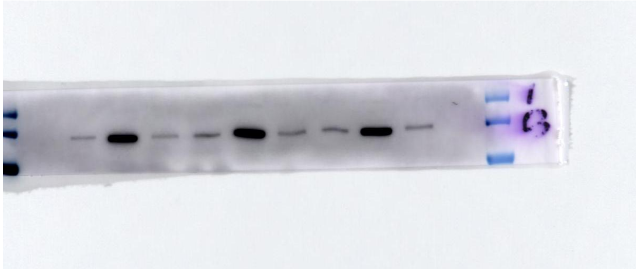

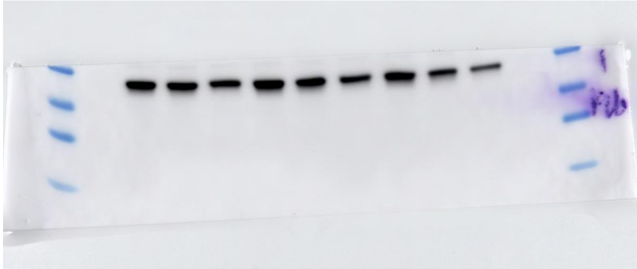

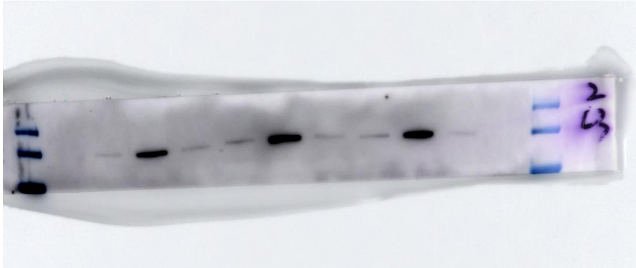

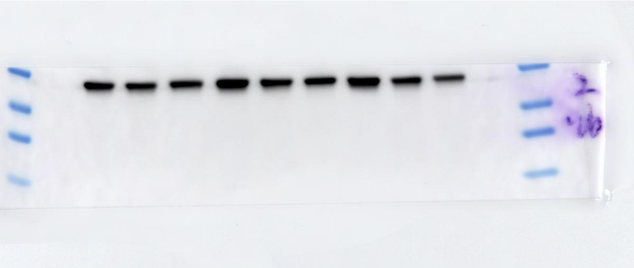

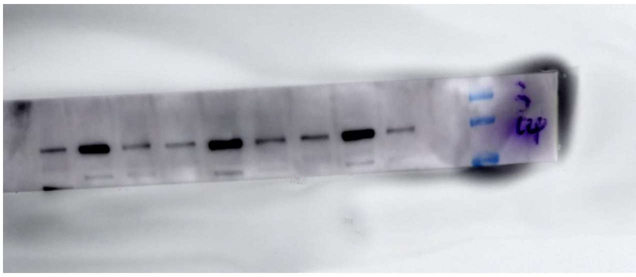

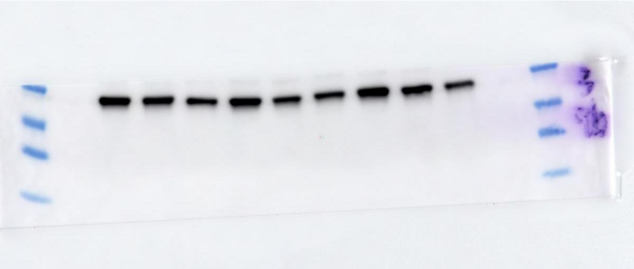

c4

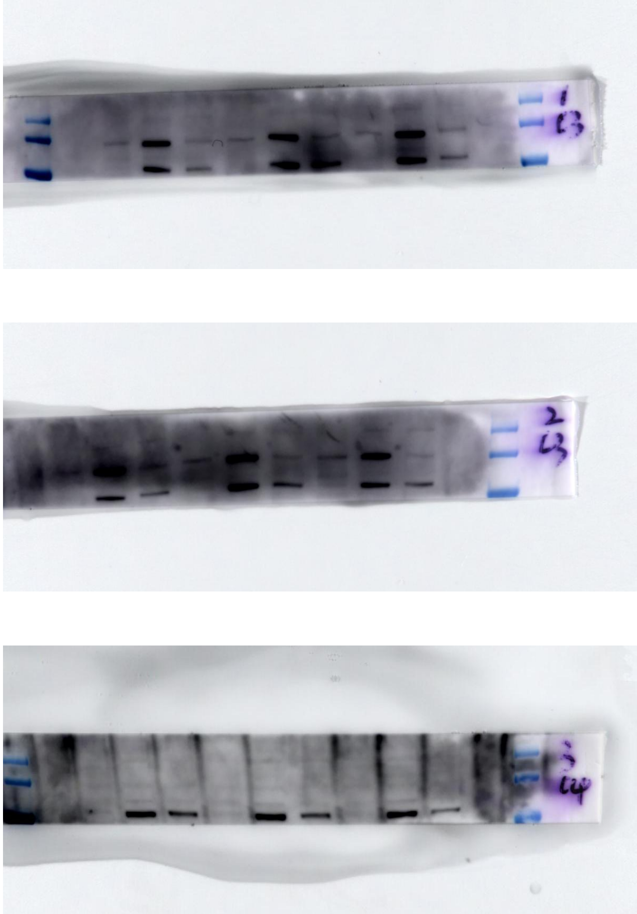

gapdh

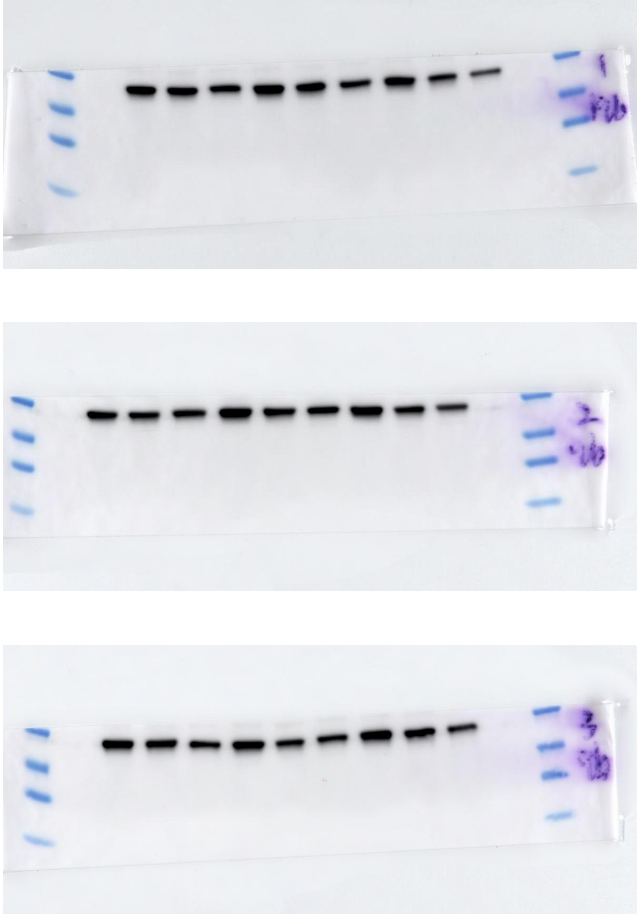

c5

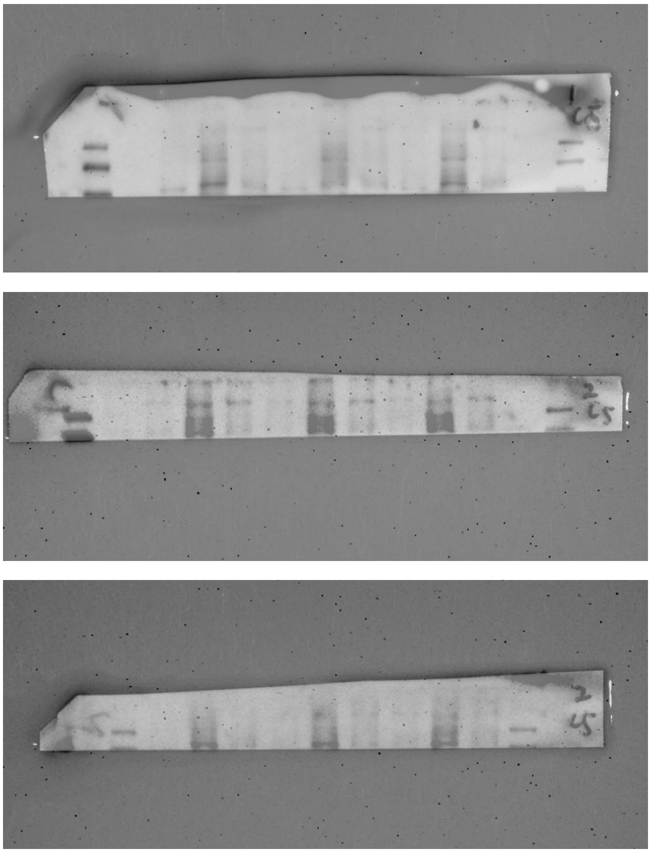

gapdh

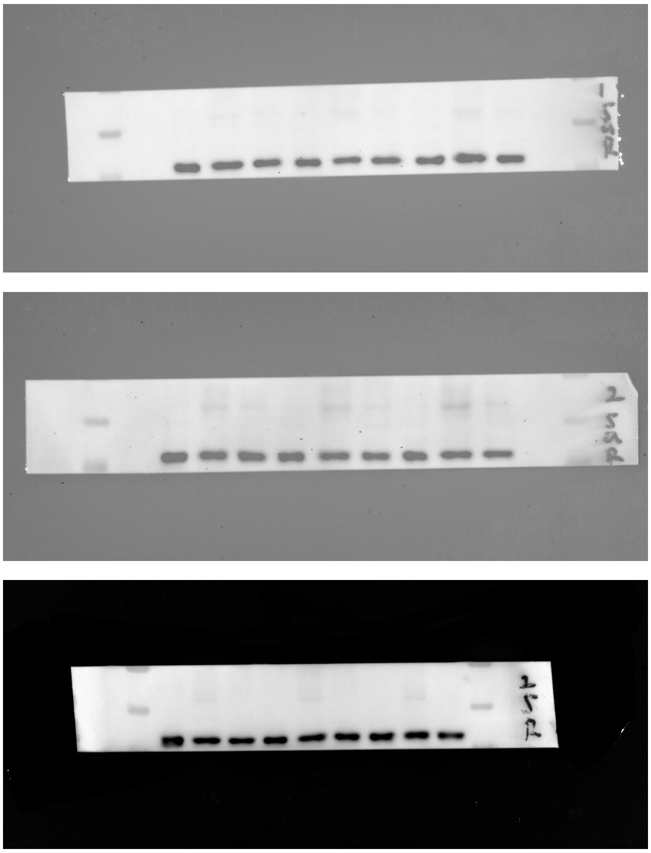

c5aR1

gapdh

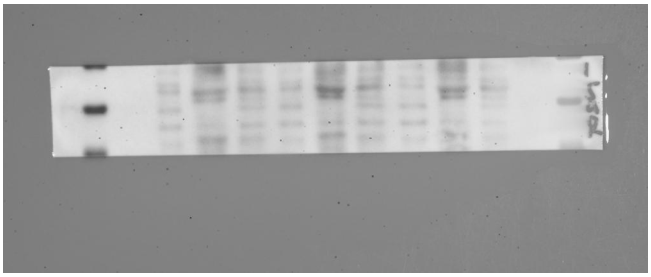

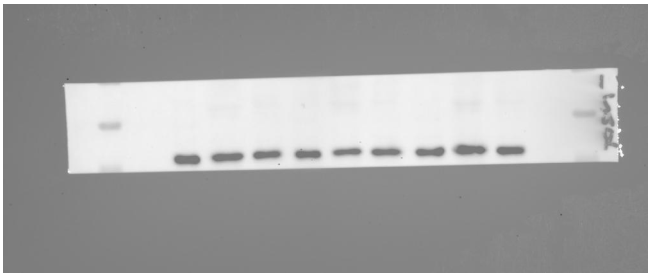

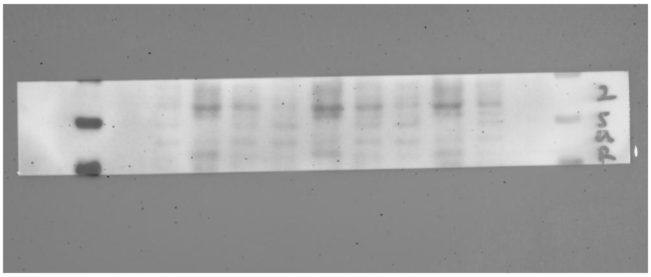

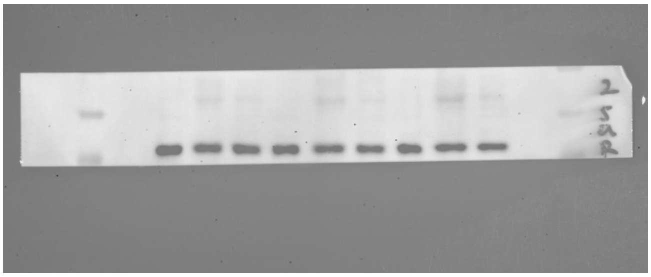

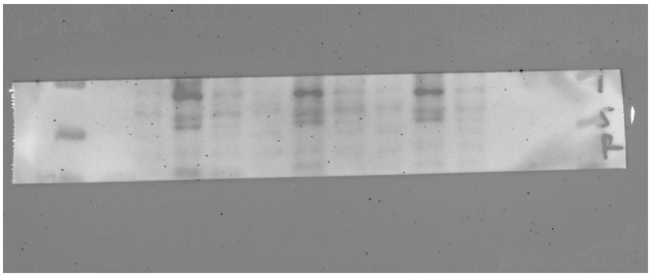

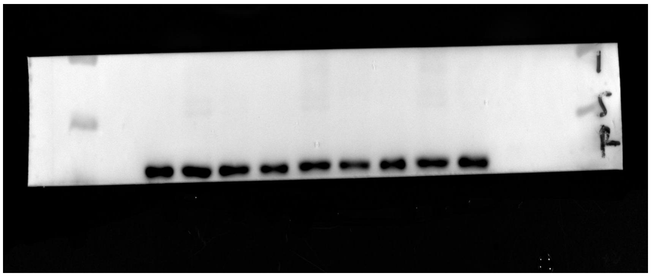

c9

gapdh

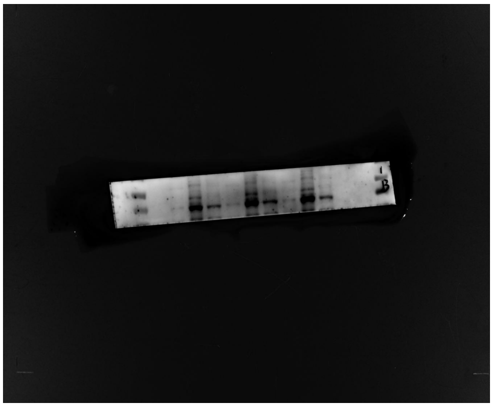

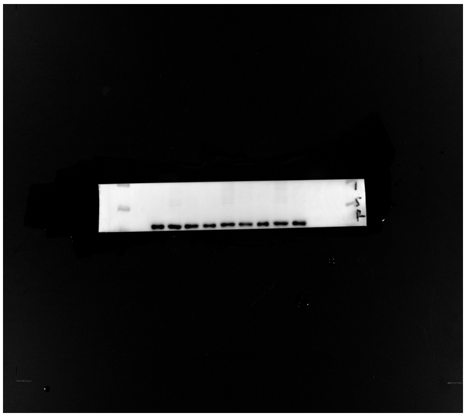

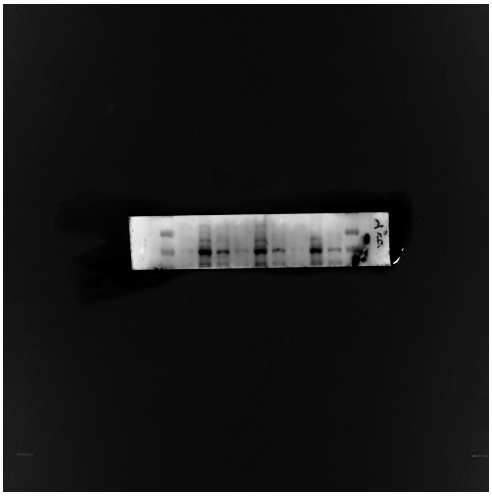

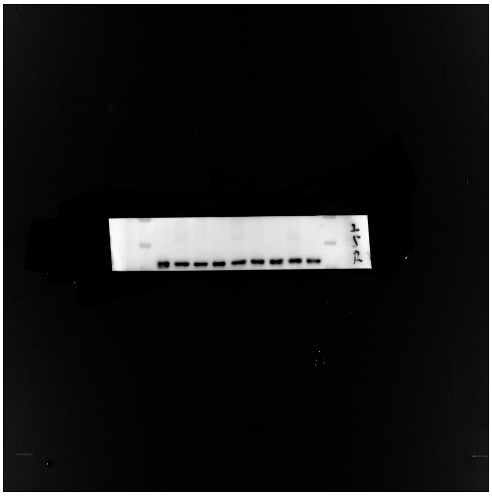

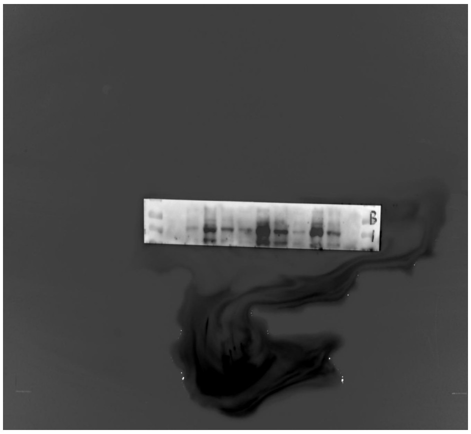

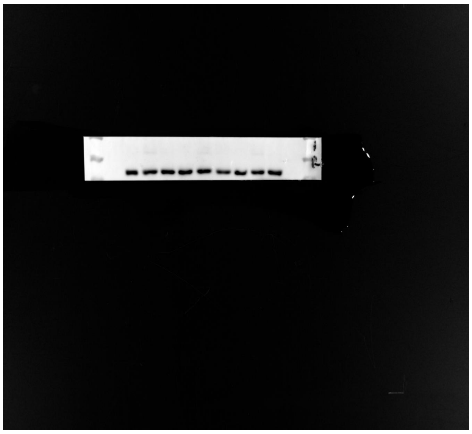

CD68

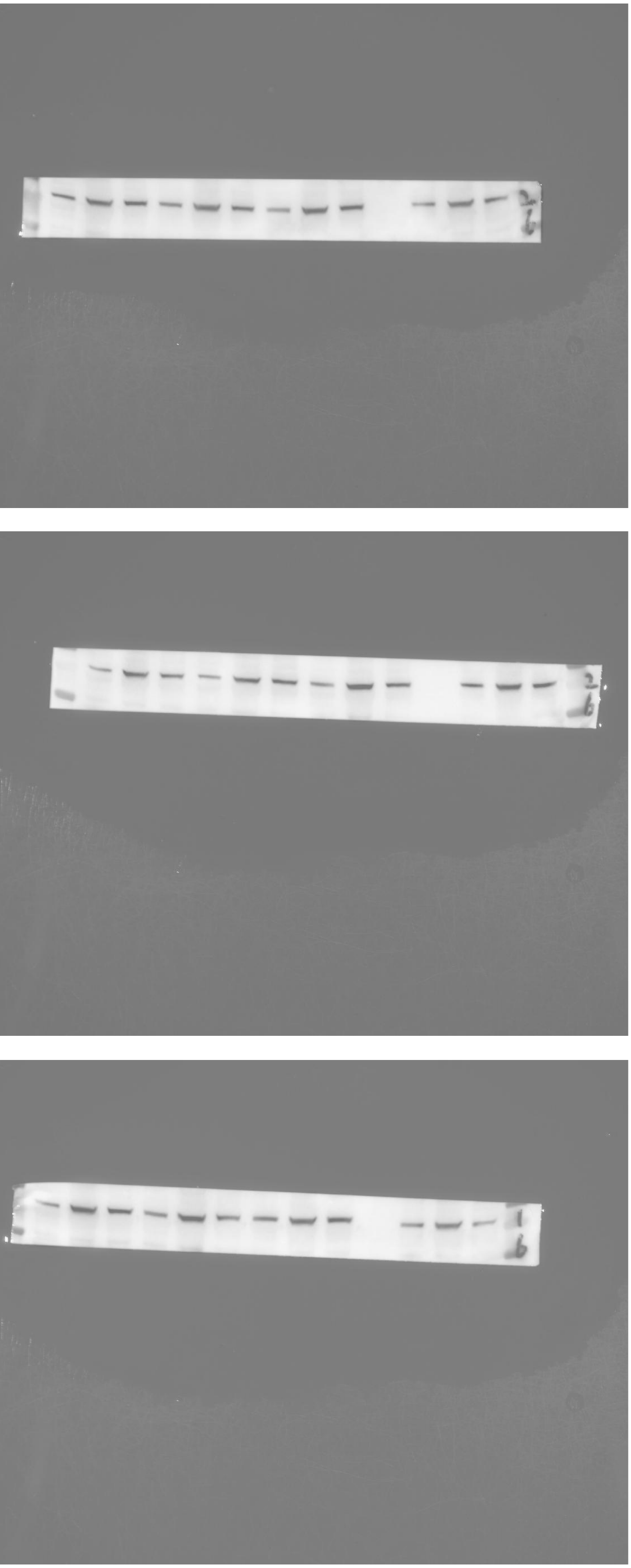

gapdh

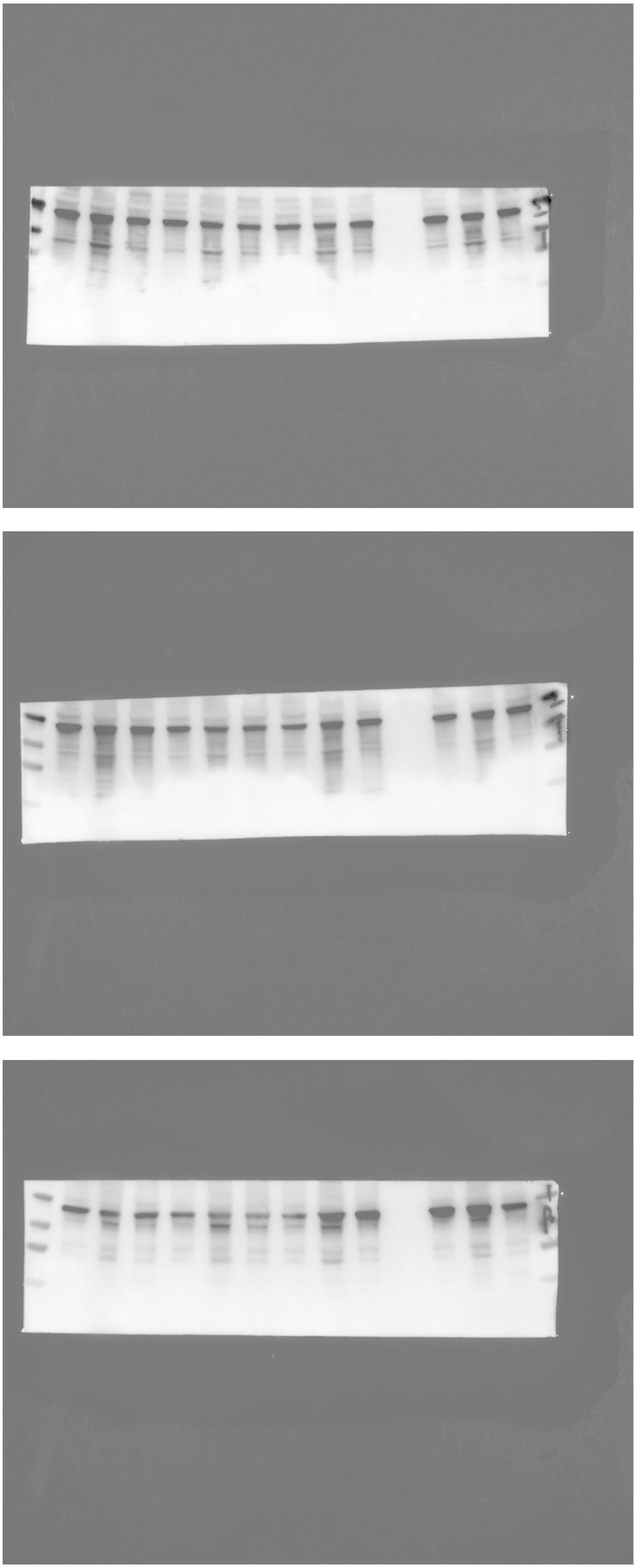

CD86 gapdh

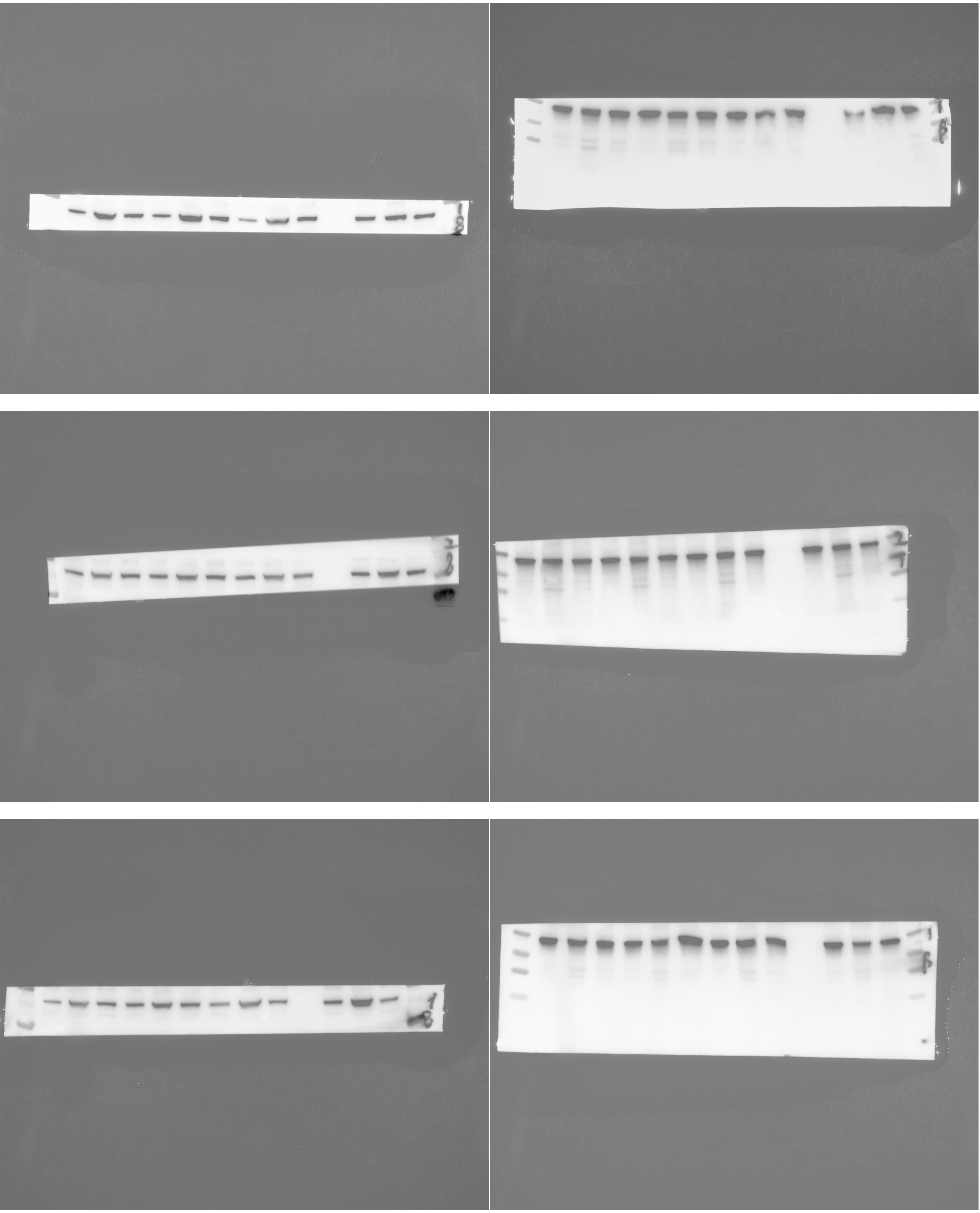

IFN-γ

gapdh

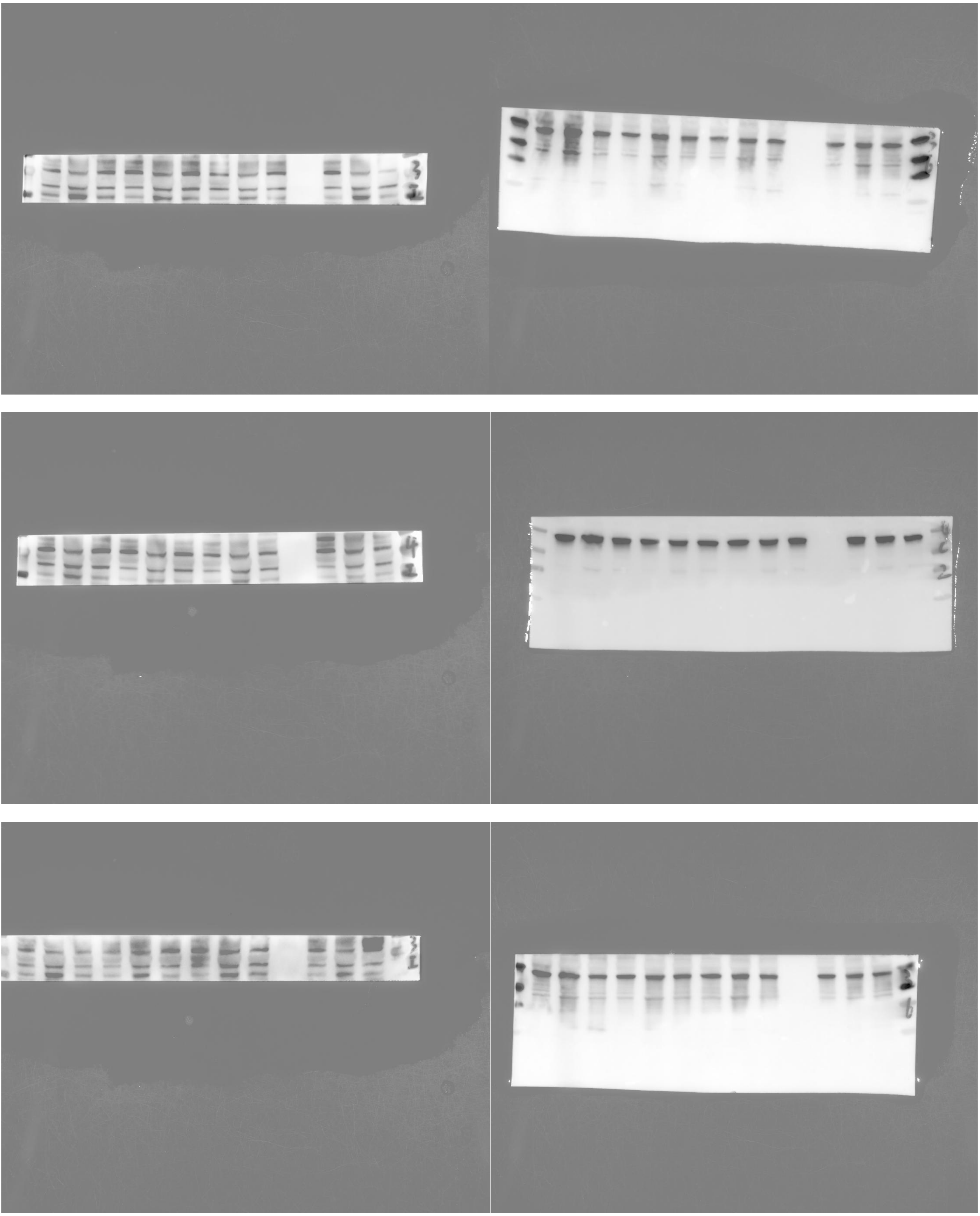

IL-1β

gapdh

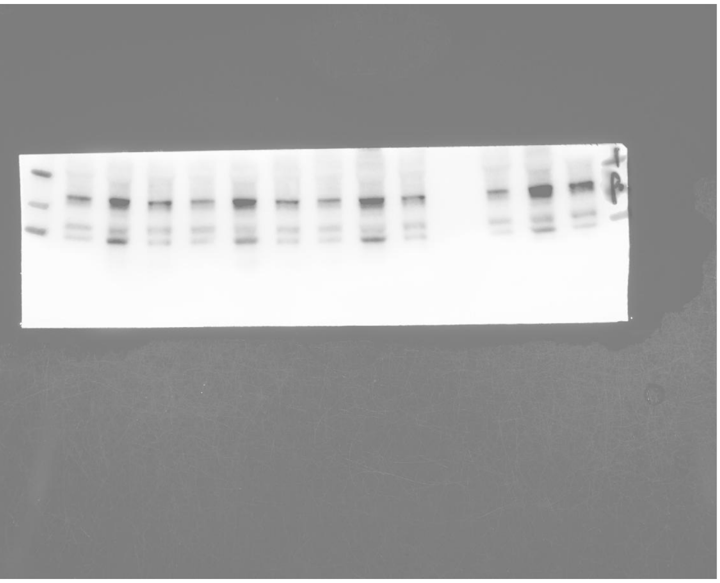

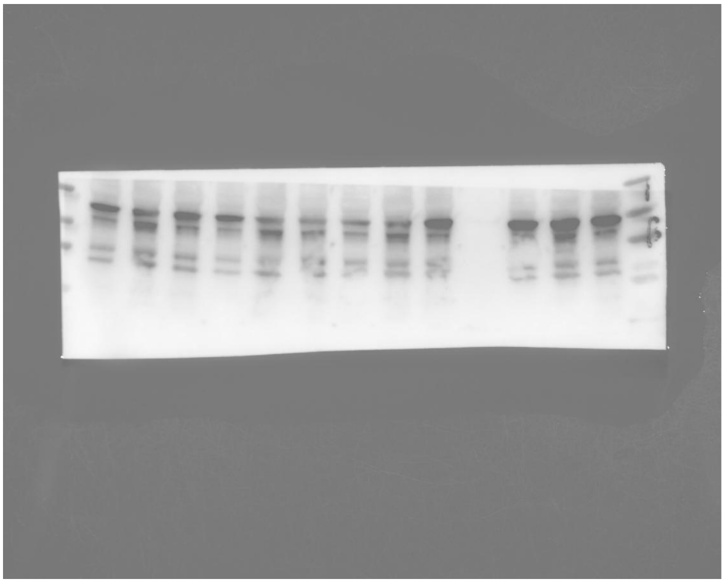

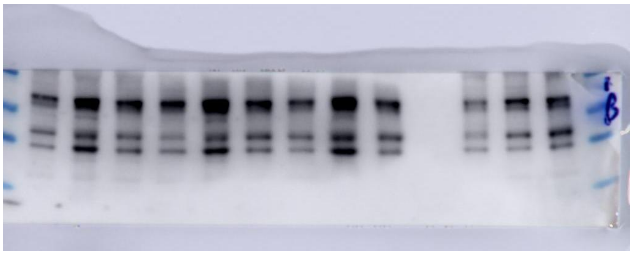

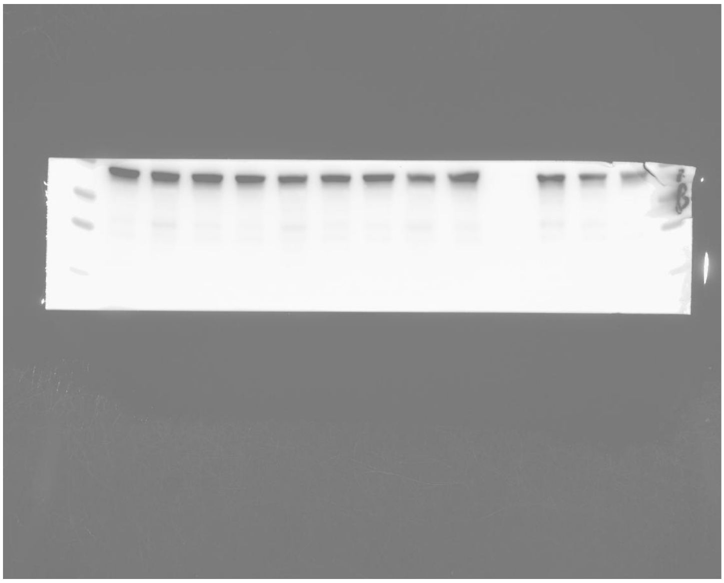

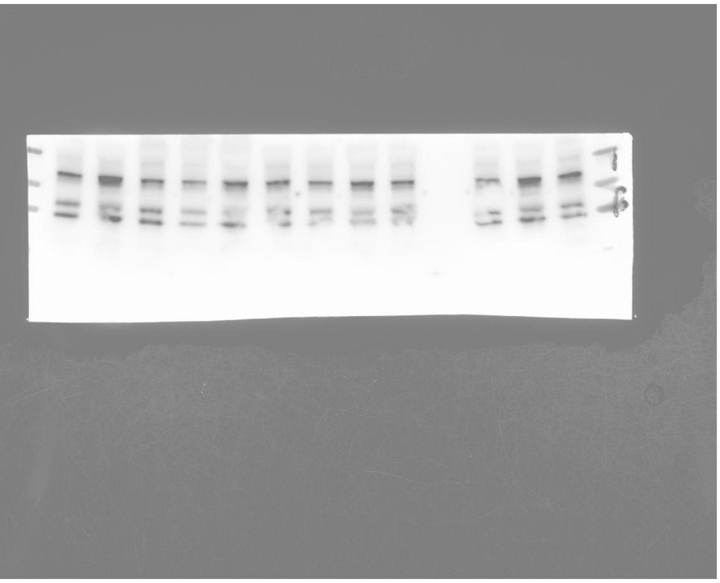

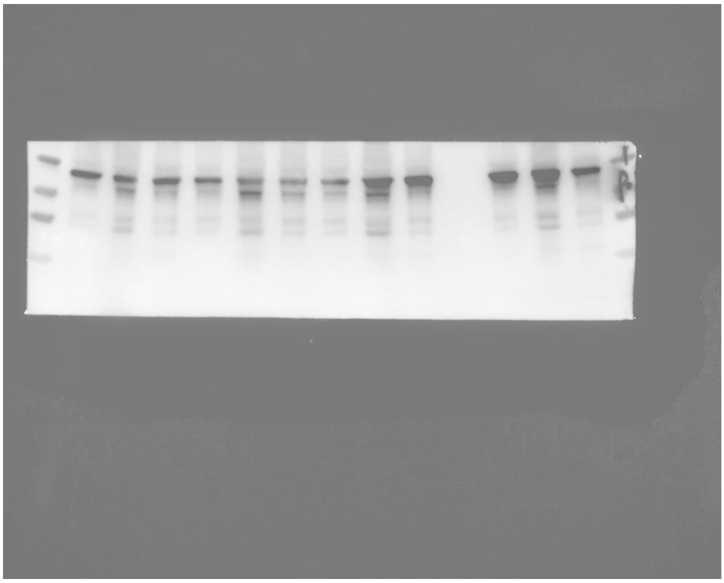

IL-6 gapdh

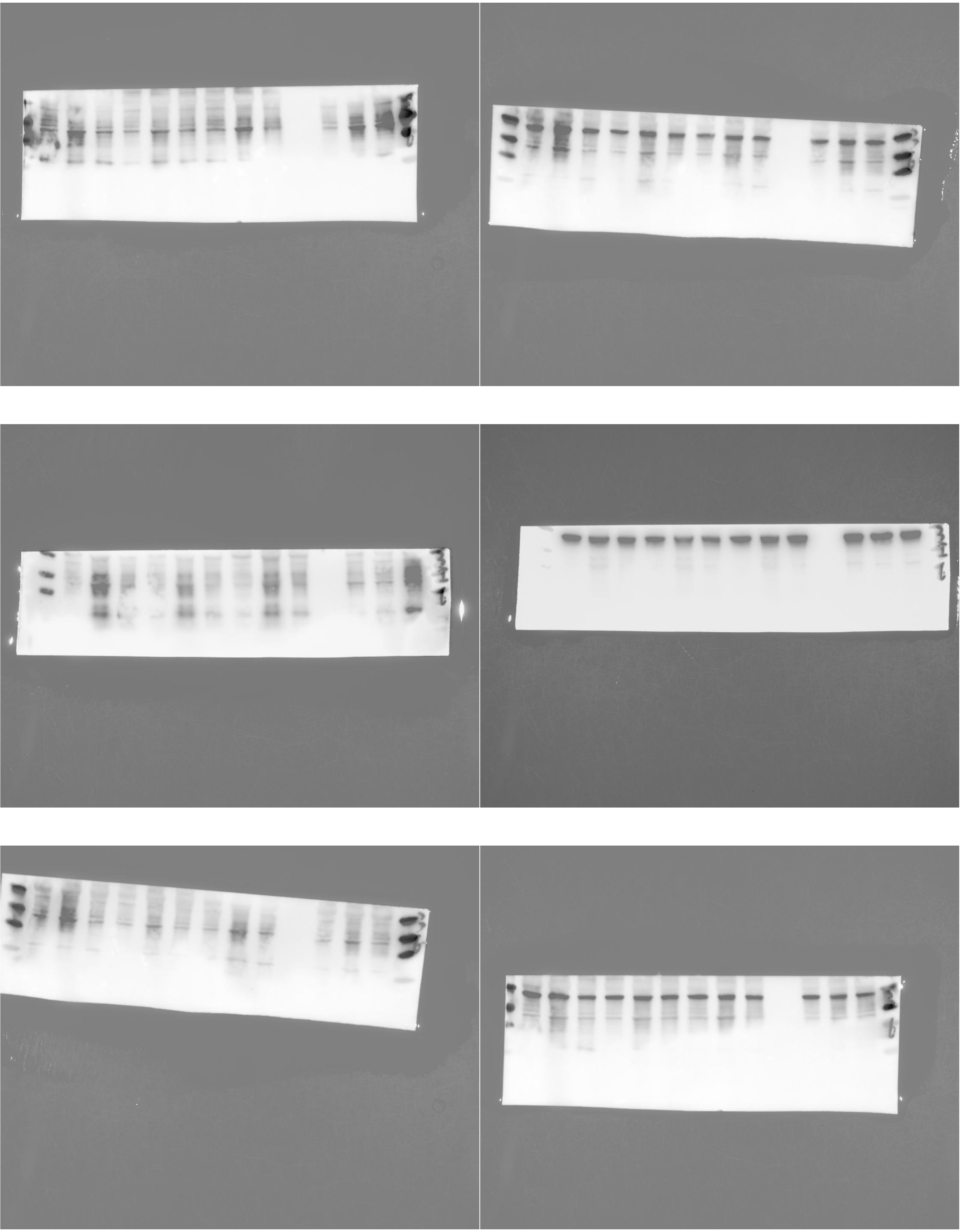

TNF-α gapdh

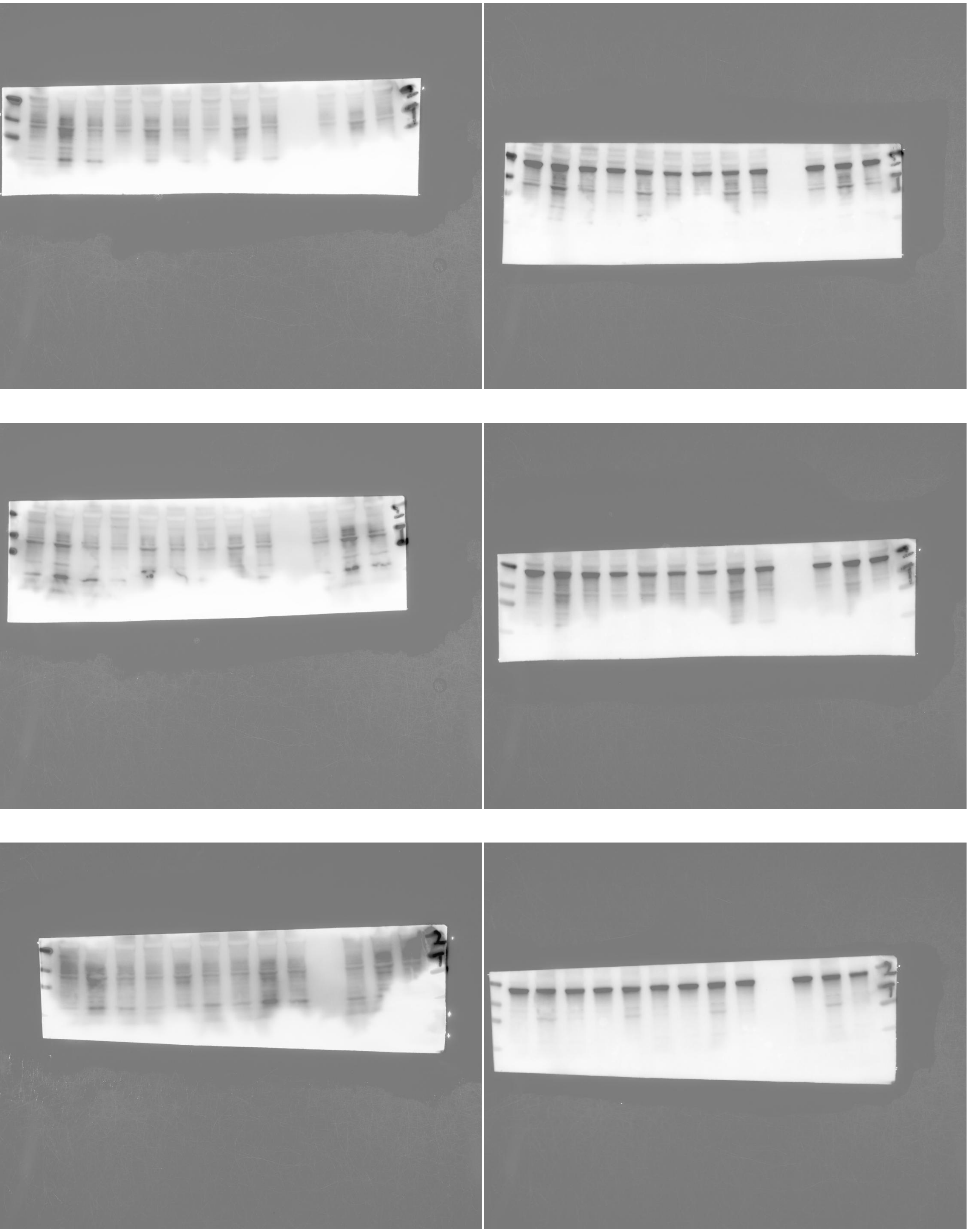

Supplement: Supplementary file 2 — Additional file 2. [file 13020_2025_1191_MOESM2_ESM.pdf]
