# Supplementary material for: Yuye Jinhua Qingre Tablets Attenuate Acute Pharyngitis by inhibiting the Complement Cascade and C5a/C5aR1 Axis
Source: Chin Med. 2025 Aug 25;20:134. doi: 10.1186/s13020-025-01191-1 (PMC12376329; doi:10.1186/s13020-025-01191-1)
Supplement: Supplementary file 3 — Additional file 3. [file 13020_2025_1191_MOESM3_ESM.docx]

| **Chinese Medicine Author Checklist** | | | |
| --- | --- | --- | --- |
| **Questions**  **(Please check the following checklist honestly, otherwise the manuscript may be rejected)** | **Yes** | **No** | **N/A** |
| **Introduction** | | | |
| 1. Is there a clear statement with background describing the hypothesis being tested by this study? | **√** |  |  |
| 2. Does the Introduction include relevant information about Chinese Medicine, such as its principles, applications, or significance in the study? | **√** |  |  |
| **Materials and Methods** | | | |
| 1. Are the sources of all materials clearly indicated? | **√** |  |  |
| 2. Is the chemical structure(s) of any compound(s) presented as a figure or referenced in the literature? | **√** |  |  |
| 3. Are the source, catalogue number, and lot for commercial antibodies indicated? | **√** |  |  |
| 4. Are the species, strain, sex, weight, and source of the animal subjects provided? | **√** |  |  |
| 5. Is the rationale provided for the selection of concentrations, doses, route, and frequency of compound/extract administration? | **√** |  |  |
| 6. Are quantified results (e.g., IC50 and/or EC50 values) of concentration- and dose-response cellular experiments included in the report? |  |  | **√** |
| 7. Is the method of anesthesia described? | **√** |  |  |
| 8. Are all group sizes approximately the same? | **√** |  |  |
| 9. Is the exact sample size (n) for each experimental group/condition clearly indicated in the text and/or on the tables and figures? | **√** |  |  |
| 10. Are the reported data displayed as the mean +/- an estimate of variability (SD, SEM) of three or more independent experimental replications? | **√** |  |  |
| 11. Is the number of replications used to generate an individual data point in each of the independent experiments clearly indicated? | **√** |  |  |
| 12. Is the threshold for statistical significance (P value) clearly indicated? | **√** |  |  |
| 13. Were post-hoc tests used to assess the statistical significance among means? | **√** |  |  |
| 14. If the study involves transcriptomics, proteomics or metabolomics, has the raw data been uploaded to public databases such as NCBI? If so, has this been stated in the manuscript? | **√** |  |  |
| 15. For human studies, has compliance with the regulations of the country(ies) where the study was conducted been explicitly stated, and has the study been registered in an accessible international registry/database (e.g., ClinicalTrials.gov, ChiCTR)? |  |  | **√** |
| 16. In case of animal studies, is there a statement indicating compliance with regulations on the ethical treatment of animals, including the **ethics approval number** and identification of the institutional committee that approved the experiments? | **√** |  |  |
| 17. Has the composition and purity of active constituents been determined and included if not from a known commercial source? | **√** |  |  |
| 1. If the study involves a compound formula or extract, has a fingerprint chromatogram been provided? |  |  | **√** |
| 19. Has the process of extraction and source of active constituents been included if not from a known commercial source? | **√** |  |  |
| 20. Has the sole effect of the vehicle used to dissolve the active constituent(s) been determined and included? | **√** |  |  |
| 21. Has conventional standard treatment been included as a positive control of efficacy? | **√** |  |  |
| **Result** | | | |
| 1. If western blots are shown, are the following included: i) appropriate loading controls, ii) replication data, iii) uncropped, original WB images should be uploaded as supplementary material. | **√** |  |  |
| 1. Were MIQE guidelines followed in the quantitative analysis and presentation of PCR and RT-PCR findings?   (MIQE, Minimum Information for Publication of Quantitative Real-Time PCR Experiments) | **√** |  |  |
| **Discussion** | | | |
| 1. Are all findings considered within the context of the hypothesis presented in the Introduction? | **√** |  |  |
| 2. Are the primary conclusions and their implications clearly stated? | **√** |  |  |
| **Conflict of Interest/Financial Support** | | | |
| 1. Is the conflict-of-interest statement included in the manuscript? | **√** |  |  |
| 2. Are all organizations providing funding for this work listed in the Acknowledgments? | **√** |  |  |
| **Figure Requirements** | | | |
| 1. Are all figures numbered and appropriately titled with descriptive legends that permit stand-alone interpretation? | **√** |  |  |
| 2. The figure layout should be neat and well-organized; no large black-and-white images; labels should be marked with a consistent font; all font size should not be smaller than 8pt; resolution should be at least 300 dpi. | **√** |  |  |
| 3. Are the whole un-cropped images of the original western blots from which figures have been derived shown as supplemental figures? (Original, unedited WB images must be uploaded.) | **√** |  |  |
| **Other Requirements** | | | |
| 1. **Does the manuscript meet the similarity index requirements: i) below 25% for the entire text, ii) no more than 20 consecutive identical words, iii) no single referenced source exceeding 5%?** | **√** |  |  |
